# Dual Cluster‐Surface Click Chemistries on Silver Nanoclusters

**DOI:** 10.1002/smsc.70289

**Published:** 2026-04-27

**Authors:** Carolina Vega Verduga, Jack E. Bowman, Jeffrey D. Henderson, Paul D. Boyle, John F. Corrigan, Mark S. Workentin

**Affiliations:** ^1^ Department of Chemistry Western University London Canada; ^2^ Surface Science Western Western University London Canada; ^3^ Department of Chemistry University of Waterloo Waterloo Canada

**Keywords:** azide, click chemistry, cluster surface, nanoclusters, silver, Staudinger–Bertozzi ligation

## Abstract

Nanoclusters’(NCs) unique chemical and photophysical properties make them among the most studied nanomaterials for applications in biosensing, catalysis, and emissive materials. Toward these, there is a need to develop NCs with ligands tuned for the desired application. This can be accomplished through direct synthesis of individual NCs or through postsynthetic reactions. Cluster surface (CS) click chemistry provides an advantageous route to produce a library of NCS from a single reactive NCs while not changing the cluster core, although examples of this are still scarce, considering the vast library of NCs. Here, the synthesis and characterization of two new azide‐functionalized Ag_20_ NCs with a carbonate template and demonstration of their cluster‐surface Staudinger–Bertozzi ligation (CS‐SBL) reactivity are reported. Furthermore, a dual‐surface double‐click strategy using CS‐SBL followed by cluster‐surface strain‐promoted azide–alkyne cycloadditions is showcased as a promising method to introduce multiple complementary functionalities under kinetic control.

## Graphical abstract

In this report, two new structures of clickable anion templated silver nanoclusters (NCs) equipped with azide functional groups capable of cluster surface click reactions are described. It is demonstrated for the first time the Staudinger–Bertozzi ligation and dual click reactions Staudinger–Bertozzi ligation‐strain‐promoted azide–alkyne cycloadditions as a strategy for postfunctionalization of silver NCs.

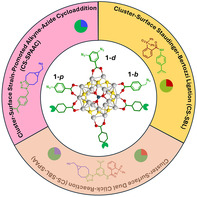

## 1 Introduction

Nanoclusters (NCs) are a sub‐nanometer material that have atomically‐precise composition and molecule‐like properties such as discrete electronic states. Noble metals like gold and silver are most commonly used to synthesize NCs, and a wide range of functional groups such as thiolates [1, 2], phosphines [3], carbenes [4, 5], alkynes [6] and carboxylates [7] can be used as stabilizing ligands. Among this group, silver NCs (AgNCs) are particularly attractive because of the lower costs of precursors and their physical properties, such as their strong luminescence [8] and potent antimicrobial properties exploited in the development of novel therapeutic agents [9].

Silver(I)‐clusters are typically synthesized by mixing silver salts and ligands in solution with additional energy input for the system from stirring, solvothermal, or ultrasonic methods [10]. A reducing agent or precursor induces the formation of anions as templates that act as structure‐directing agents [11], influencing the size, shape, and stability of the resulting AgNCs [12]. The most common templates are inorganic oxyanions that bear different charges and geometry. The carbonate anion (CO_3_^2−^) is the most common templated anion, and it can be generated in situ from atmospheric CO_2_ [13]. The core of Ag(I) ions can be further stabilized by a variety of ligands through a self‐assembly synthetic strategy.

Development of high‐nuclearity AgNCs depends not only on the inorganic template but also on the silver precursor, solvent, and type of ligand [11]. Self‐assembly is a common approach for synthesizing structure‐defined silver(I) clusters. For example, Sun and coworkers [14] synthesized a drum‐like Ag_20_ core NCs [(CO_3_)@Ag_20_(S^t^Bu)_10_(DMF)_6_(NO_3_)_8_] with tert‐butylthiolates and nitrates as stabilizing ligands. This cluster was shown to be susceptible to further transformation by replacing the weakly bound nitrates with more strongly coordinating ligands. This approach was explored by Zang and coworkers [15] who demonstrated this shell‐ligand nitrate substitution without altering the Ag_20_ core, using carboxylic acids such as benzoic acid, ferrocene carboxylic acid and alrestatin to substitute all 8 nitrates on the [(CO_3_)@Ag_20_(S^t^Bu)_10_(DMAc)_4_(NO_3_)_8_] with carboxylate ligands (R‐CO_2_−), generating new functionality to introduce fluorescence or electrochemical activity. Previous work from our group focused on the preparation of CO_3_@AgNCs with *m‐* and *p‐*azidobenzoate ligands [(CO_3_)@Ag_20_(S^t^Bu)_10_(CO_2_‐*m/p*‐C_6_H_4_‐N_3_)_8_(DMF)_4_] [16], where we demonstrated that the azides could participate in cluster‐surface strain‐promoted azide–alkyne cycloadditions (CS‐SPAAC) to introduce new functionality on all 8 of the azides without changing the Ag_20_NC core structure. An additional example of the application of the SPAAC reaction includes Rück and coworkers, who had applied this strategy to conjugate DNA‐stabilized Ag_16_ NC with three different peptides [17]. This postsynthetic modification of AgNCs is a desirable property accessible through the addition of ligands with pendant functional groups. Other work from He et al. demonstrated the introduction of chirality to an Ag_21_NC with pendant aldehydes through aldimine condensation in high yields [18]. Further work that develops the toolbox of chemistry accessible at the cluster‐surface is necessary to expand the scope, selectivity, and complexity of transformations that can be performed.

The Staudinger–Bertozzi ligation (SBL) is a bioconjugation method in which a covalent linkage is created through an amide bond formation [19]. This bioorthogonal click reaction was first reported by Bertozzi and coworkers [20], using azido‐sugars treated cells and a biotinylated phosphine with a methyl ester electrophilic trap. SBL exhibits slow reaction kinetics that allow the real‐time quantification of the system either by the release of an emissive probe or the formation of phosphine oxide [21]. The synthesis of phosphine‐based reagents required for the SBL involves fewer steps than that of strained alkynes and can be easily modified to introduce different groups at the ester site or on the aromatic ring. Applications of the Staudinger–Bertozzi ligation include medicinal chemistry and in vivo labeling [22]. SBL applications have also emerged in the materials sciences, such as the functionalization of nanoparticles and polymers, because of its chemoselectivity and the formation of a stable amide bond. The interfacial SBL of triarylphosphine‐functionalized gold nanoparticles [23] with azide was demonstrated and characterized through ^31^P{^1^H} NMR spectroscopy and X‐ray photoelectron spectroscopy (XPS). Using a similar approach, the controlled release of rhodamine B from gold nanoparticles was accomplished through SBL [24] as well as double‐click reactions on the AuNP surface [25]. To date, the SBL has not been demonstrated in Ag or Au NCs.

In this work, two new azide‐bearing Ag_20_NCs were obtained through a one‐pot synthetic approach with 3,5‐diazidobenzoic acid and 4‐(azidomethyl)benzoic acid, and their postsynthetic functionalization through click chemistry was demonstrated. The ligand 3,5‐diazidobenzoate was selected to have additional reactive sites compared to mono‐azido functionalized ligands; the ligand with the azide group in the benzylic position was selected expecting to increase the reaction rate toward SBL in comparison to phenyl azido ligands [26]. The cluster‐surface Staudinger–Bertozzi ligation (CS‐SBL) is demonstrated on AgNC for the first time as an additional type of cluster surface (CS) bioorthogonal click chemistry (CS‐BCC). Because each AgNC carries multiple azides, the combination of SBL and SPAAC for a dual‐click reaction is also demonstrated. Employing a ligand with multiple azide groups on a NC allows adding multi‐functionality either with different complementary CS‐BCC reaction partners or through two different CS‐BCC reactions.

**SCHEME 1:**
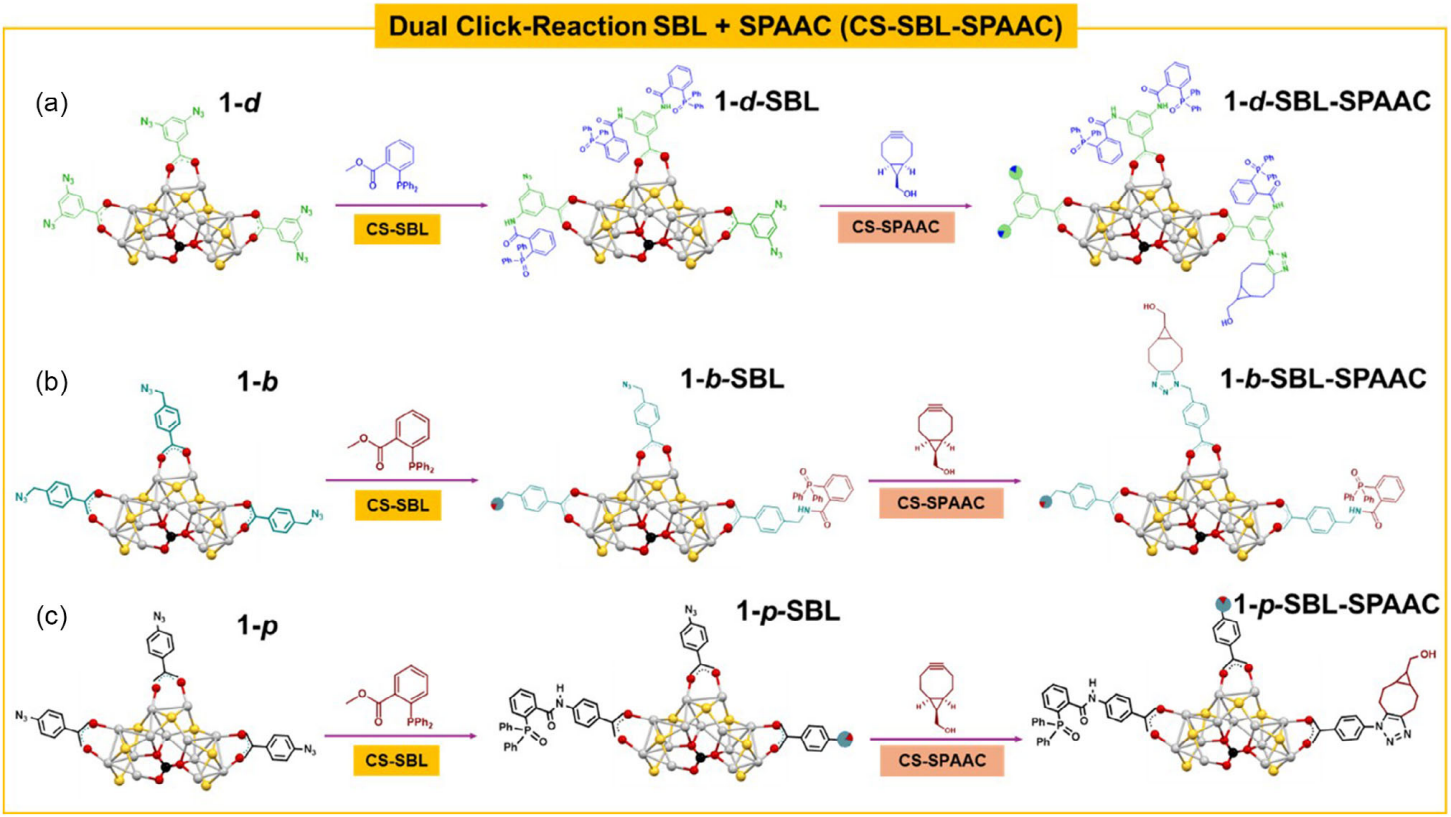
Representation illustrating CS reactions, CS‐SBL and subsequent CS‐SPAAC reaction, taking place on (a) cluster **1–*d*** (b) cluster **1–*b*** (c) cluster **1–*p***. Only a portion of the CS is shown.

Two novel NCs, including 3,5‐diazidobenzoate derived [(CO_3_)@Ag_20_(S^t^Bu)_10_(3,5‐N_3_‐C_6_H_3_COO)_8_(DMAc)_4_] (**1–*d***) and 4‐(azidomethyl)benzoate (benzylazido) functionalized [(CO_3_)@Ag_20_(S^t^Bu)_10_(p‐(N_3_‐CH_2_)‐C_6_H_4_COO)_6_(NO_3_)_2_(DMAc)_4_] (**1–*b***) are reported. Importantly, proof of concept CS‐SBL and a dual click reaction (SBL + SPAAC) was demonstrated with cluster **1–*d***, **1–*b*** and p‐azidobenzoate derived AgNC **1–*p*** (Scheme 1).

Additionally, in this work, a detailed analysis of the CS‐SPAAC reaction performed on the clusters **1–*d*** and **1–*b*** with BCN is included (Figures 2 and 3). Synthesized clusters as well as clicked products were fully characterized with Nuclear Magnetic Resonance (NMR), fourier‐transform infrared (FT‐IR) spectroscopies and further examined using X‐ray photoelectron spectroscopy (XPS) and X‐ray absorption spectroscopy (XAS). The latter are shown to be powerful tools to track the CS chemistry on NCs. The cluster structures (**1–*b*** and **1–*d***) were verified by single‐crystal X‐ray diffraction (SCXRD).

## 2 Results and Discussion

Throughout, abbreviations are used to refer to specific Ag_20_ NCs. The NC synthesized with 3,5‐diazido benzoate as the ligand is referred to as **1–*d*** (*d* for di). When 4‐(azidomethyl)benzoate is the ligand, the NC is designated **1–*b*** (for azide in the **b**enzylic position), and the cluster bearing 4‐azidobenzoate as the ligand is referred to as **1–*p*** (*p‐for para*).

Syntheses of Ag_20_NC **1–*b*** and **1–d** were accomplished using a modified version of a previously reported protocol [15]. In short, silver tert‐butylthiolate and silver nitrate were dissolved in either DMAc:ACN or DMF:ACN; after the complete dissolution of the silver precursors, the ligand was added to the solution, and single crystals were obtained after slow evaporation of the solvent within 2 days. The ligands 3,5‐diazidobenzoic acid and 4‐(azidomethyl)benzoic acid were used as capping ligands for **1–*d*** and **1–*b***, respectively. Ligand synthesis was done according to previously reported protocols covered in detail in the SI. Some advantages of clusters **1–*b*** and **1–*d*** are that they can be obtained in scalable and useable quantities, > 100 mg scale was typical, as colorless block crystals with yields above 75% and in under 3 days at normal laboratory conditions.FT‐IR (Figures S1‐S3) was used to confirm the presence of the azide functional group as part of the ligands in clusters **1–*d*** and **1–*b***. For cluster **1–*d***, the azide asymmetric stretching is at 2112 cm^−1^ and for cluster **1–*b*** at 2086 cm^−1^.

NMR spectral analysis (^1^H and ^13^C{^1^H}) (Figure S4–S8) of both clusters (**1–*b*** and **1–*d***) gave insight into cluster composition. The ^1^H NMR spectrum (**.**) of **1–*d*** shows a doublet at 7.59 ppm and a triplet at 6.72 ppm that corresponds to the aromatic protons of the carboxylate ligands. The tert‐butylthiolate (S^t^Bu) signal appears as a singlet at 1.73 ppm and coordinating solvent signals (DMAc) can also be observed at 3.02 ppm, 2.95 ppm and 2.09 ppm. The integration ratio of the aromatic:DMAc:S^t^Bu proton signals for **1–*d*** is consistent with a composition of eight carboxylate ligands, four coordinating solvent molecules, and ten tert‐butylthiolate ligands, which means 24 aromatic protons (16 + 8), 36 solvent protons (12 + 12 + 12), and 90 S^t^Bu protons. The structure was confirmed by SCXRD.

Similar analysis of the ^1^H NMR spectrum (Figure S6) can be done for the cluster **1–*b***, where two sets of doublets can be found in the aromatic region that corresponds to the aromatic protons of the carboxylate ligand. A singlet at 4.42 ppm corresponds to benzylic hydrogens, and solvent signals (DMAc) can also be accounted. The cluster **1–*b*** obtained from the solvent mixture DMAc:ACN have six carboxylate ligands instead of eight like the cluster **1–*d***; two nitrate groups located at top and bottom of the drum‐like core offer additional stabilization to this six‐substituted system. As an additional attempt to obtain the eight‐substituted version of cluster **1–*b***, the synthesis was also carried out using a 1:1 mixture of DMF and ACN instead of DMAc. The recovered crystals exhibit increased solubility in chloroform, the integration of the^1^H NMR resonances (Figure S8) supports the formation of an eight‐substituted cluster bearing eight carboxylate ligands. A suitable structural description of this NC could not be obtained via SCXRD due to sample inhomogeneity and poor crystal quality; for this reason, we did not continue with this derivative.

Analysis of S^t^Bu signal is almost at the same chemical shift (singlet at 1.67 ppm), for cluster **1–*b*** synthesized either with DMF or DMAc as solvent. Similar behavior of the S^t^Bu resonance had been observed with Ag_20_ NC synthesized with different regioisomers such as *p*‐azidobenzoic (singlet at 1.66 ppm) acid and *m*‐azidobenzoic acid [16]. Comparison between **1–*b*** and **1‐*d*** indicates that the S^t^Bu resonance is affected by the nature of the azido ligand, where the S^t^Bu resonance is slightly upfield at 1.67 ppm for **1–*b*** relative to **1–*d***, consistent with weak electron‐withdrawal from aryl azides for **1–*d***.

**FIGURE 1:**
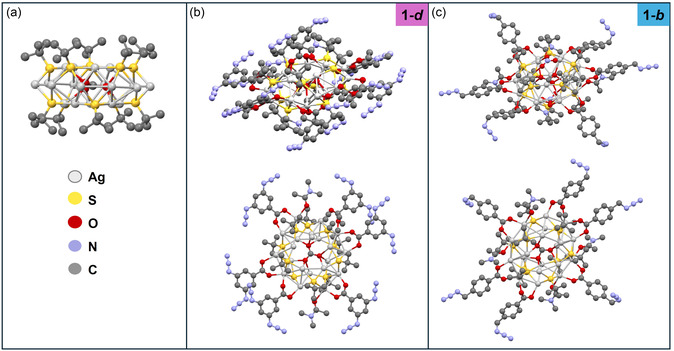
Molecular structure of (a) Ag_20_ core of NC (carboxylate ligands are omitted for clarity) (b) cluster [(CO_3_)@Ag_20_(S^*t*^Bu)_10_(3,5‐N_3_‐C_6_H_4_COO)_8_(DMAc)_4_] (**1‐*d***) side view and front view (top and bottom carboxylates omitted for clarity) (c) cluster [(CO_3_)@Ag_20_(S^*t*^Bu)_10_(*p*‐(N_3_‐CH_2_)C_6_H_4_COO)_6_(NO_3_)_2_(DMAc)_4_] (**1‐*b***) side and front view (top and bottom nitrates omitted for clarity).

Single crystals suitable for SCXRD analysis were obtained after 2 days of slow evaporation of the solvent at room temperature in the dark. **1–*d*** crystallizes as colorless prisms in the triclinic space group *P*−1 (Figure 1b) and possesses a Ag_20_ core with ten bridging S^t^Bu ligands similar to the previously described clusters (Figure 1a) and a carbonate ion in the center that acts as a template [14, 15]. The sample crystal was a nonmerohedric twin and was indexed to two domains, the twin fraction was refined to a value of 0.4139(6); the structure exhibits a variety of disorders as well as highly disordered solvent. The Ag(I)‐‐‐Ag(I) distances range from 2.9553(9) Å to 3.3700(2) Å in indicating attractive argentophilic interactions [27] and the Ag–S bond distances are in the range of 2.4001(19) Å – 2.6400(2) Å. Crystals obtained for **1–*b*** also crystallize in the triclinic space group *P*−1 (Figure 1c). The Ag–S bond distances range from 2.388(4) Å to 2.6442(19) Å, while the Ag(I)‐‐‐Ag(I) distances range from 2.9225(14) Å to 3.327(12) Å. The molecular structure exhibited several disorders: as typical for this class of compound, the templating CO_3_^2−^ anion is disordered across a crystallographic center of symmetry, and the nitrate anion is disordered over two orientations.

The cluster cores of **1–*b*** and **1–*d*** share the same composition and exhibit similar bond distances to one another. These features are also comparable to the previously described cluster **1–*p***, which depicts a surface decorated with *p*‐azidobenzoate [16], as well as the eight‐substituted nitrate cluster [14] and confirm the minimal impact of the ligand substitution on the core size of this type of cluster. In addition to the different ligands used in their synthesis, another key distinction lies in the number of carboxylate ligands present in each cluster. In cluster **1–*d***, all eight nitrate ions are replaced with carboxylate ligands, while cluster **1–*b*** contains only six carboxylates, with two nitrate ions remaining as part of the structure.

**FIGURE 2:**
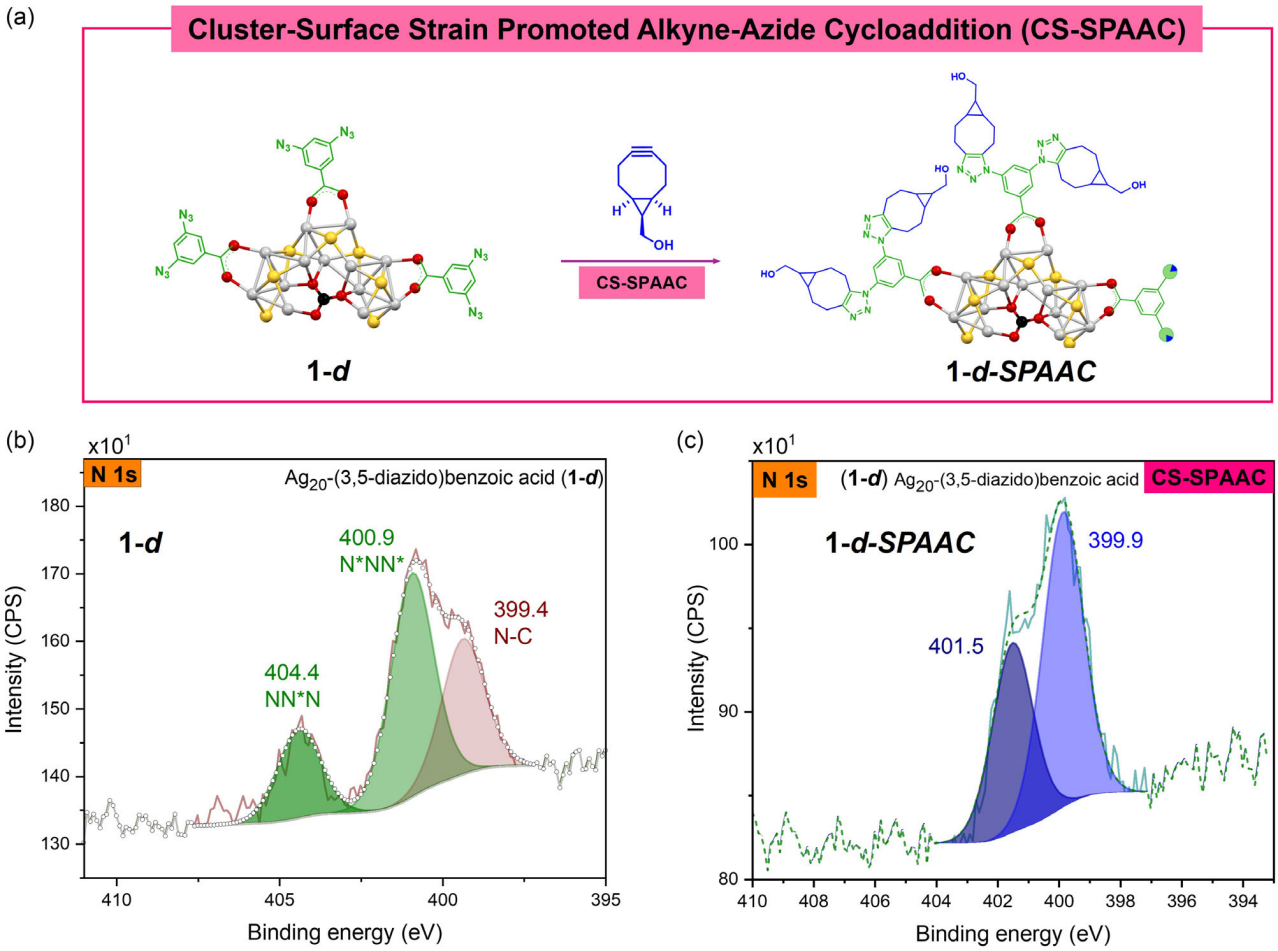
(a) Representation illustrating the CS strain promoted alkyne‐azide cycloaddition (CS‐SPAAC) taking place on cluster **1–*d***. N 1s high‐resolution spectra of (b) **1–*d***, 404.4 eV and 400. 9 eV corresponding to N_3_ and 399.4 eV assigned to N‐C (c) **1–*d*–SPAAC**, 401.0 eV and 399.6 eV assigned to triazole (N = N, N‐C).

XPS was used to further characterize the cluster and the subsequent CS‐BCC reactions. The survey spectrum of cluster **1–*d*** (Figure S9a) shows the expected elements carbon, oxygen, nitrogen, sulfur and silver; atomic percent value of most components is related to the molecular formula of the cluster, except for C 1s due to adventitious carbon and N 1s due to degradation of azide during the data collection. The high‐resolution spectra of each element were also collected (Figure S9a); it can be noticed from the O 1s and C 1s high‐resolution spectra the components associated with the carbonyl, carboxylate and thiolate groups that are part of the NC structure. The binding energy of the Ag 3d_5/2_ at 368.8 eV and the value of Auger parameter (Table 1) are indicative of the oxidized silver present in the core [28]. The binding energy of S 2p_3/2_ at 162.9 eV is in agreement with thiolates on metal surfaces like silver [29]. The high‐resolution N 1s spectrum of **1–*d*** (Figure 2b) was fitted with three components, two of which (404.4 eV and 400.9 eV) are attributed to the azide functional group. The component at 399.4 eV is attributed to the N‐C from the solvent molecules and from the known induced degradation of azido groups during XPS analysis [30].

**FIGURE 3:**
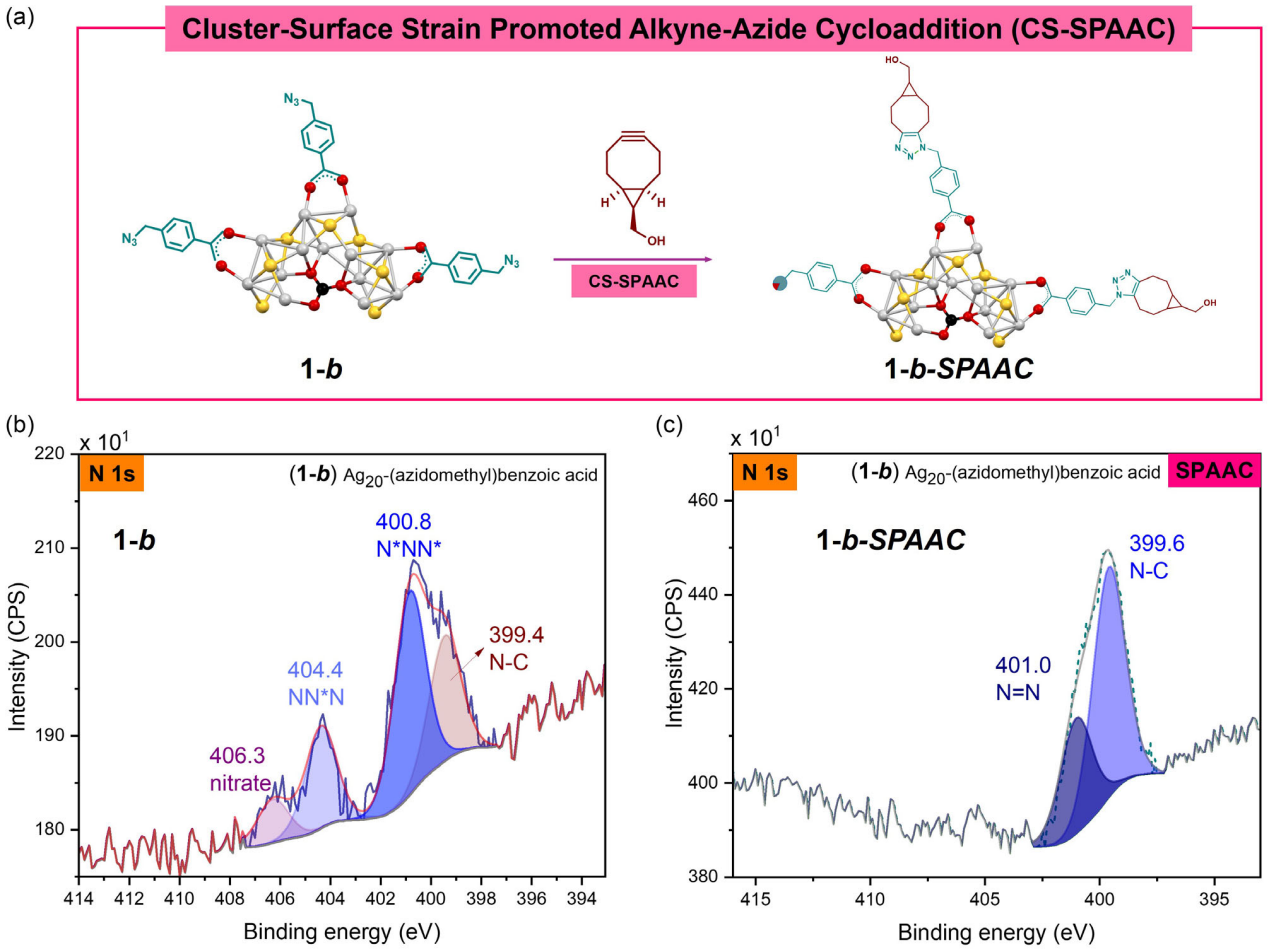
(a) Representation illustrating the CS strain promoted alkyne‐azide cycloaddition (CS‐SPAAC) taking place on cluster **1–*b***. N 1s high‐resolution spectra of (b) **1–*b***, 406.3 eV assigned to nitrate, 404.4 eV and 400.8 eV corresponding to N_3_ and 399.4 eV assigned to N‐C (c) **1–*b*–SPAAC**, 401.0 eV and 399.6 eV assigned to triazole (N = N, N‐C).

Analysis of the survey spectrum of cluster **1–*b*** (Figure S10a) confirms the absence of impurities in the material and the high‐resolution spectra of each element were obtained (Figure S10). The high‐resolution Ag 3d spectrum was deconvoluted into components related Ag 3d_5/2_ and Ag 3d_3/2_, the peaks were fitted with the same full width half maximum (FWHM) and spin–orbit separation of 6.0 eV [28]. The analysis of the Auger parameter (Table 1) is consistent with the presence of oxidized Ag; for instance, the Ag_20_ core is formed through the self‐assembly process of Ag(I) ions from the silver precursors. The high‐resolution O 1s spectrum was fitted with three components due to the oxygen from different functional groups in the NC. From the SCXRD and ^1^H NMR spectral data, the NC has six carboxylate ligands, two nitrate ligands, one templating carbonate anion and four solvent molecules (DMAc); these considerations were taken into account for the area constraint in the fitting. Without this supporting information, modeling of the O 1s spectra would have been difficult due to the significant amount of overlap between species [31]. The component at 530.7 eV is assigned to the oxygens in the carboxylate ligand [32], the component at 531.6 eV is due to the oxygens in both the carbonate [33, 34] and nitrate ligands [34] and the component at 532.4 eV is assigned to the carbonyl oxygen in the molecules of DMAc [35]. The high‐resolution N 1s spectra (Figure 3b) demonstrates four components, two of them (404.4 eV and 400.8 eV) are attributed to the azide functional group. The component at 406.3 eV is assigned to the nitrate that acts as a ligand. The component at 399.4 eV is attributed to the N‐C from the solvent molecules and the known induced degradation of azido groups during XPS analysis [30].

These azido‐NC can be added to the Ag_20_ NC library for CS‐SPAAC and expand the library for different CS‐BCC. In this work, two different click reactions were tested as proof of concept with each cluster (**1–*d*** and **1–*b***). The CS‐SPAAC (strain‐promoted alkyne azide cycloaddition) with the model alkyne *endo*‐bicyclo [6.1.0]non‐4‐yn‐9‐ylmethanol (BCN) gave as product the corresponding triazole **1–*d*–SPAAC** (Figure 2a) and **1–*b*–SPAAC** (Figure 3a). The *endo*‐BCN diastereomer was selected over the *exo*‐BCN because it is slightly more reactive in the SPAAC reaction and only one was used to simplify the spectral characterization. The progress of the reaction was monitored by tracking changes in the azide moiety using FT‐IR spectroscopy, where the azide stretching band at 2100 cm^−1^ gradually decreased in intensity until it was no longer detectable in the final clicked product. Despite multiple recrystallization attempts, suitable crystals for SC‐XRD analysis of the clicked products could not be obtained. Therefore, NMR, XPS and XAS were employed to confirm the composition of the clicked NC. Detailed analysis of the XPS high‐resolution N 1s spectra of the starting NC and the clicked products confirms the successful surface modification. Figures 2 and 3 show the high‐resolution N 1s spectra for **1–*d***, **1–*b*** and clicked products.

After the CS SPAAC reaction (**1–*b*** + BCN_endo_), changes in the survey, O 1s (Figure S11) and N 1s high‐resolution (Figure 3c) spectra offered confirmation that the reaction has proceeded. The O 1s high‐resolution spectrum was fitted with two components, the component at 531.1 eV is attributed to carboxylate. The component at 532.4 eV could be attributed to the C = O from the DMAc, carbonate, and the hydroxyl group from the BCN‐OH. In the N 1s high‐resolution spectra (Figure 3c) the broad signal can be fitted with two components having binding energies of 401.0 eV and 399.6 eV [36], with a 1:2 ratio of the integrated area that corresponds to the three nitrogen atoms in the triazole group. Additional confirmation that the click reaction has proceeded is the absence of the azide signal at 404.4 eV. FT‐IR spectrum of the clicked product (Figure S2) shows evidence of azide consumption during the click reaction. Analysis of the ^1^H NMR spectrum for (**1–*b*** + BCN_endo_) (Figure S12) shows a downfield shift of the benzylic protons from 4.42 ppm in CDCl_3_ to 6.64 ppm in DMSO‐*d*_6_, attributed to the triazole formation and integration of the signals is indicative that the reaction goes to completion.

Analysis of the CS SPAAC reaction (**1–*d*** + BCN_endo_) product using same spectroscopic techniques showed complete azide consumption and triazole formation. FT‐IR spectrum of the clicked product shows the disappearance of the azide stretching frequency and a new broad signal at 3300 cm^−1^ associated with the incorporated ‐OH of the alcohol handle on the BCN strained alkyne (Figure S1). The ^1^H NMR spectrum for (**1–*d*** + BCN_endo_) (Figure S13) shows a downfield shift of the aromatic protons from the carboxylate ligand attributed to the triazole formation. Integration of the two aromatic signals and the signal at 4.29 ppm related to the methylene group outside the BCN ring have a ratio of 2:1:4, which is indicative that both azides in the carboxylate ring had reacted completely with BCN_endo_. The XPS N 1s high‐resolution spectrum of the clicked product (Figure 2c) shows an absence of the azide peaks and two new signals at 401.5 eV and 399.9 eV consistent with the nitrogen atoms of the triazole group [37]. Additional XPS analysis (Figure S14) corroborates the CS–SPAAC reaction.

**TABLE 1:** Binding energy and Auger parameter for the initial NC and the clicked products.

Component	**Ag 3d**_**5/2**_ **, BE**	**Ag M**_**4**_ **N**_**45**_ **N**_**45**_ **, KE**	**AP‐3d**_**5/2**_ **, M**_**4**_ **N**_**45**_ **N**_**45**_
**1–*b***	368.6	355.7	724.2
**1–*b*** CS**–**SPAAC	368.4	356.2	724.6
**1–*b*** CS**–**SBL	368.4	355.8	724.2
**1–*b*** CS**–**SBL**–**SPAAC	368.5	355.4	724.0
**1–*d***	368.8	355.0	723.8
**1–*d*** CS**–**SPAAC	368.4	356.1	724.6
**1–*d*** CS**–**SBL	368.6	355.8	724.4
**1–*d*** CS**–S**BL**–**SPAAC	368.7	355.7	724.4
**1–*p*** CS**–**SBL	368.5	355.8	724.3

Expanding the scope of CS‐BCC, the Staudinger–Bertozzi ligation (SBL) was tested with the azide functionalized clusters **1–*b***, **1–*d*** and ***1*–*p*** using 2‐diphenylphosphanyl benzoic acid methyl ester as a model phosphine (Scheme 1). In an SBL reaction, azides react with the phosphorus center to generate an iminophosphorane intermediate, which subsequently reacts with the carbonyl group to form a ligation via amide bond formation, with phosphine oxide present on the ligated product. CS**–**SBL reactions were carried out on 60 mg scale of each respective cluster, reacted with an excess of SBL phosphine in CH_2_Cl_2_ with added H_2_O, and stirred overnight. CS**–**SBL click products were isolated the next day by centrifugation in a CH_2_Cl_2_/hexanes mixture. Accordingly, analysis of phosphorus environments by XPS and ^31^P{^1^H} NMR spectroscopy confirmed that the SBL reaction occurred on the CS.

**FIGURE 4:**
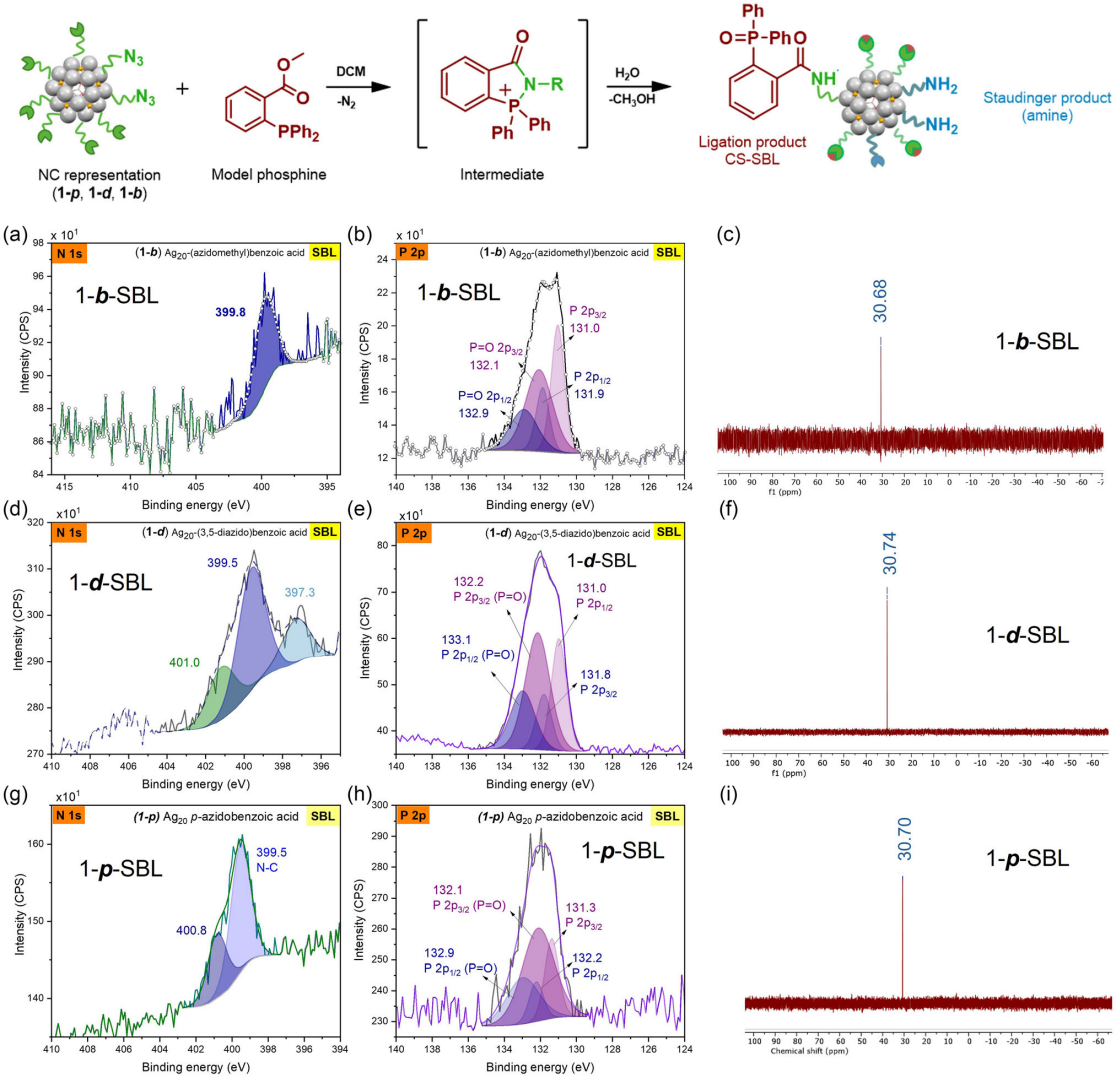
Characterization of the ligated products **1–*b*–SBL** (a) N 1s high‐resolution spectrum (b) P 2p high‐resolution spectrum (c) ^31^P{^1^H} NMR, **1–*d*–SBL** (d) N 1s high‐resolution spectrum (e) P 2p high‐resolution spectrum (f) ^31^P{^1^H} NMR spectrum and **1–*p*–SBL** (g) N 1s high‐resolution spectrum (h) P 2p high‐resolution spectrum (i) ^31^P{^1^H} NMR spectrum.

The XPS survey spectrum of the clicked product **1–*b*–SBL** shows the expected elements (Ag, C, S, O, P, N) without impurities (Figure S15a). The P 2p high‐resolution spectrum was fitted with four components (two species) Figure 4b. The P 2p_3/2_ and P 2p_1/2_ doublet for each chemical species is constrained to have a 2:1 peak area ratio, equal FWHM and a peak separation of 0.86 eV. The signal P 2p_3/2_ at 132.1 eV [23] can be assigned to the *P* = O, the additional peak at 131.0 eV can be related to labile coordinating phosphine and other phosphorus‐containing species in the system. The signal S 2p_3/2_ at 162.0 eV is in agreement with thiolates on metal surfaces like silver [29]. The O 1s high‐resolution spectrum was fitted with three components with equal FWHM; the fitting reveals an increase in the area of the component at 532.0 eV related to the additional amide and *P* = O formed after the click reaction, the component at 533.7 eV can be associated with an ester. The N 1s high‐resolution spectrum (Figure 4a) was fitted with a single signal at 399.8 eV (N‐C), the intensity of the signal is associated with the reduced amount of N in the clicked product.

The system **1–*b*–SBL** bearing only one azide per ligand and a methyl group serves as a good model for the analysis of the CS reaction, where the multiplicity of the benzylic protons signal changed from a singlet to a doublet due to the coupling with the N‐H from the amide group formed. Amide protons are expected to be found downfield, but factors like hydrogen bonding and solvent can influence the chemical shifts. The ^1^H NMR spectrum for the cluster **1–*b*–SBL** is shown in Figure S16, where signals at 9.13 ppm and a doublet at 4.15 ppm are evidence of the amide formed as the ligation product. The four sets of doublets that can be found in the aromatic region correspond to the aromatic protons of the ligated and the nonligated forms of the carboxylate ligands. The broad signal at 1.63 ppm can be assigned to the protons of the tert‐butylthiolate, these ligands contribute to holding the Ag_20_ core together. The accurate integration of the signals was complicated due to the overlapping signals of aromatic protons from the triaryl phosphine present, which adds complexity to the system. However, focusing on NMR regions with less overlap, the proton integration for the carboxylate ligand and the thiolate align well with the NC description and support that the cluster core's integrity is not compromised during the ligation. The observed ^31^P{^1^H} NMR resonance at 30.7 ppm (Figure 4c) is attributed to the *P* = O formed in the ligated product.

The SBL reaction rate can be influenced by the solvent polarity, the phosphine substituents, and the azide structure [26]. For example, during the last step of the reaction (hydrolysis step), the yield of the ligation product is influenced by the sterics of the ester leaving group of the phosphine. When the ester leaving group is more sterically demanding, the hydrolysis of the aza‐ylide yields both the ligation product (amide) and the hydrolyzed product or Staudinger product (amine) [26]; in the latter case, the phosphine oxide is not part of the molecule.

The cluster **1–*d*** and the model phosphine were also reacted together, where the ligated product **1–*d*–SBL** exhibits the resonance at 30.7 ppm in the ^31^P{^1^H} NMR spectrum (Figure 4f). The ^1^H NMR (Figure S17) shows a broad signal at 1.58 ppm, which is assigned to the tert‐butylthiolate, proper assignment of the signals in the aromatic region became demanding, but the signal at 10.59 ppm reflects amide formation; it was interesting to notice a broad signal at 3.64 ppm that is evidence that the hydrolysis step has also formed amine as product, that was later confirmed with after the analysis of the simpler system **1–*p***. The partial azide consumption was confirmed with the reduced intensity of the IR stretching. The fitting analysis of the N 1s high‐resolution XPS spectrum (Figure 4d) reveals signals at 401.0 eV and 399.5 eV associated to C–N and =N‐ from amide, amine, and azide degradation product; the peak at 397.3 eV is indicative of a P‐N [37] interaction, which confirms the stabilization of the SBL intermediate on the CS. Analysis of the P 2p high‐resolution XPS spectrum (Figure 4e) confirms the presence of *P* = O. Additional XPS spectra of the system **1–*d*–SBL** are depicted in Figure S18.

The XPS analysis of **1–*p*** (Figure S19) revealed the addition of P upon the reaction from the survey spectrum as well as the additional elements (Ag, C, S, O, N) present in the NC. The P 2p high‐resolution spectrum (Figure 4h) has peak at 132.9 eV associated with the *P* = O, peaks at 399.5 eV and 400.8 eV in the N 1s high‐resolution spectrum (Figure 4g) as well as FT‐IR spectrum confirms that azide was consumed (Figure S3). The ^1^H NMR spectrum for **1–*p*–SBL** (Figure S21) shows 6 distinct doublets in the aromatic regions, which were attributed to three ligand species at different points in the SBL reaction progress, being unreacted azide, Staudinger product, and Staudinger–Bertozzi ligation product. Relative integration of these peaks revealed that 2.5 out of 8 azido ligands on average remained unreacted after the reaction, while of the remaining 5.5 ligands, 3 were hydrolyzed to form amines and 2.5 were present as the phosphine oxide product. Longer reaction times of up to 5 days revealed no significant difference in the degree of ligation, indicating that further reaction on the CS is not an issue of slow reaction kinetics. The findings in the ^1^H NMR spectrum were consistent with that of the ^31^P{^1^H} NMR spectrum, which showed a peak at 30.7 ppm corresponding to phosphine oxide (Figure 4i). IR spectral analysis further supported the conclusion of a partial reaction on the cluster‐surface, where a reduction in the intensity of azide asymmetric stretching frequency at 2113 cm^–1^ was observed (Figure S3).

The CS‐SBL reaction proceeded more slowly on the NC surface and did not reach completion for both **1–*b***, **1–*d***, and **1–*p*** compared to the corresponding reactions with small organic molecules. Although cluster **1–*b***, bearing benzyl azide, was expected to react more rapidly than cluster **1–*d***, which contains phenyl azide [26], due to the electron‐donating effect of the benzyl group, the CS ligation still required extended reaction times, typically involving overnight stirring at room temperature. Analysis of the systems **1–*d*** and **1–*p*** reveals that the hydrolysis step yields amine formation (Staudinger product), and the aromatic azide promotes the stabilization of SBL intermediates, preventing them from hydrolyzing. Given the steric hindrance introduced by the triaryl motif and the intrinsically slow kinetics of the SBL, incomplete conversion evidenced by residual azide is anticipated.

In a subsequent attempt to drive the reaction to completion, the mixture was stirred for several days. However, this prolonged exposure led to changes in the NC, likely due to interactions between phosphorous and the silver cluster core, as evidenced by a broad signal at 12.60 ppm in the^31^P{^1^H} NMR spectrum. This assignment was confirmed by performing the SBL reaction with a non‐azide functionalized cluster (benzoate‐Ag); the cluster functionalized with benzoic acid reacted with the model phosphine and purified using the same conditions as for Ag_20_‐N_3_. Analysis of the ^31^P{^1^H} NMR spectrum of the product (Figure S22) reveals a broad signal at 12.4 ppm due to Ag‐P interactions and a small signal at 31.0 ppm assigned to the oxidized form of the model phosphine.

One of the benefits of having multiple azides on a AgNC (or indeed on a single ligand) is that it opens the possibility to perform two or more CS‐BCC reactions in a single NC. In the present case, the presence of unreacted azide groups following Staudinger–Bertozzi ligation on the NC surface presented an easy opportunity to investigate these dual‐click reactions (Scheme 1) and to probe their modification based on the different reaction rates. To target the remaining azides after the CS‐SBL, the click CS‐SPAAC partner BCN was introduced into the reaction mixture, leveraging the rapid kinetics of the SPAAC reaction and the lower steric demand. Two click chemistries (SBL and SPAAC) were performed consecutively on the CS. Clusters **1–*d***, **1–*b*** and **1–*p*** were reacted with the model phosphine overnight and in the same pot BCN was added; the purification process was done through hexanes precipitation. The obtained SBL‐SPAAC products have reduced solubility. Complete assignment and integration of the NMR signals of these double‐clicked products is complex but the^1^H‐NMR spectra for **1–*d*–SBL–SPAAC** (Figure S23), **1–*b*–SBL–SPAAC** (Figure S24) and **1–*p*–SBL–SPAAC** (Figure S25) reveals that resonances related to phosphine and BCN are part of the product. The ^31^P{^1^H} spectra (Figure S26–S28) shows signal attributed to *P* = O at 28.1 ppm

The XPS analysis of **1–*d*–SBL–SPAAC** (Figure S29) and **1–*b*–SBL–SPAAC** (Figure S30) reveals a changes in atomic percentage from the different compositions of these samples; for example, values obtained from the survey spectrum of **1–*b*–SBL–SPAAC** are in agreement with a cluster where three ligands reacted toward SBL ligation and three triazole rings from the SPAAC reaction are formed. This assignment agrees with the integration of the benzylic protons from the NMR spectrum. Additional elements C, P, N, O, S, and Ag have the expected binding energies, analysis of the Auger parameter provides similar values as the parent clusters and one‐click systems. Additional analysis of this dual‐click product was performed with XAS where the FT‐EXAFS of the Ag K‐edge (Figure S31–S32) shows comparable bond scattering paths as for the initial clusters **1–*d***, **1–*b*** and **1–*p***.

Attempts to provide additional information on the molecular composition of the initial clusters **1–*b***, **1–*d***, **1–*p***, showed extensive fragmentation likely due to the labile nature of silver‐carboxylates. Despite this, analysis of the clicked and double‐clicked by ESI‐MS was achieved by assigning some of the peaks found in the mass spectra to expected ligand fragments, as shown in Table S1. The expected ligand fragments are indicative of SBL ligation product, Staudinger product and triazole formation.

**FIGURE 5:**
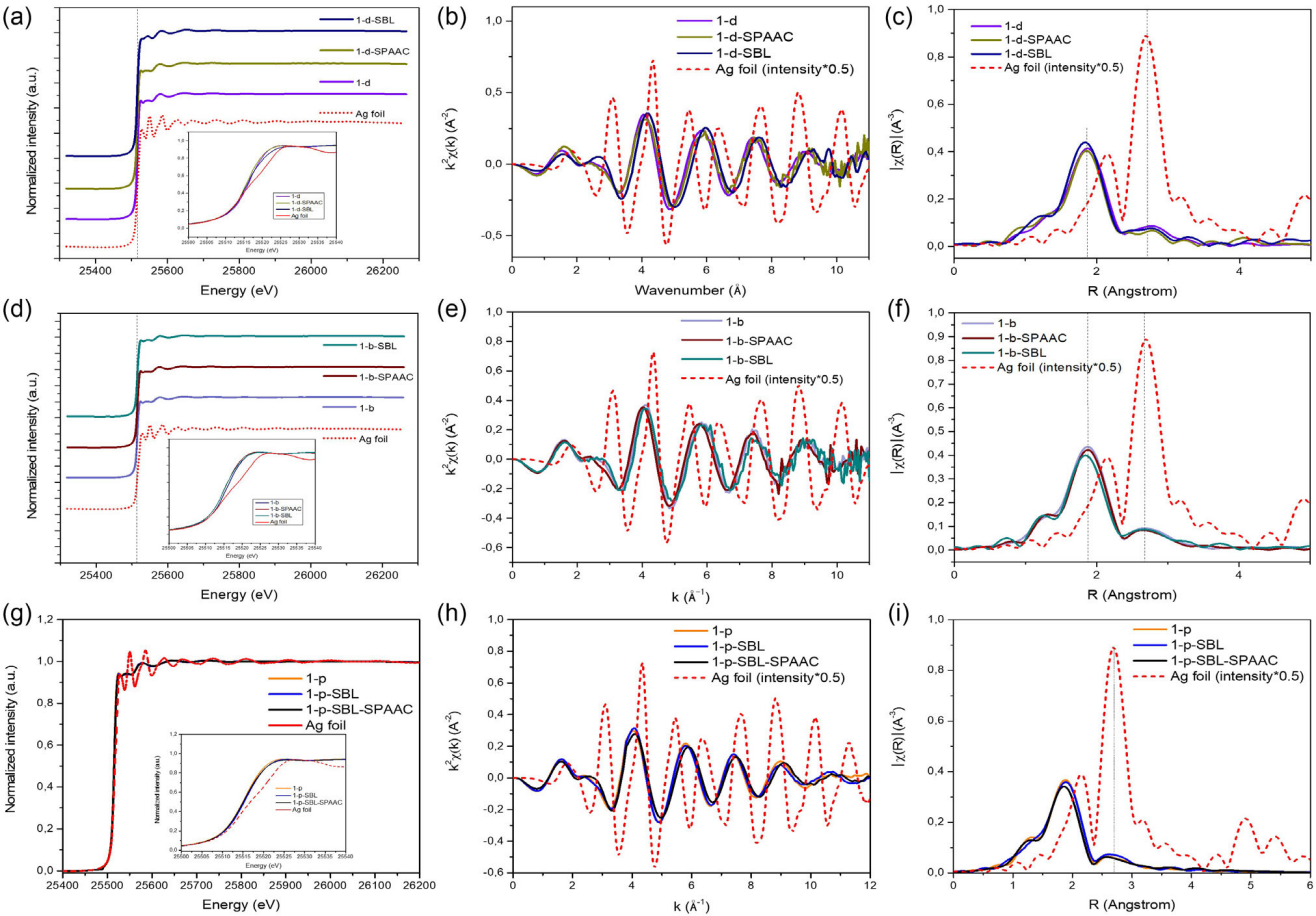
Ag K‐edge XAS spectra with an inset of the XANES region (a) **1‐*b*** and clicked products (d) **1‐*d*** and clicked products (g) **1‐*p*** and clicked products. Ag K‐edge FT‐EXAFS shown in K‐space and R‐space of (b) (c) **1‐*b*** and clicked products (e) (f) **1‐*d*** and clicked products (h) (i) **1‐*p*** and clicked products.

The reactions between the ligands on the Ag_20_NC and the click partners have been successfully demonstrated by different spectroscopic techniques. However, to probe the effect of the changes in the ligand environment on the NC core needs further investigation. For this reason, synchrotron XAS was used to probe the structure of the NC at an atomic scale. Analysis of the Ag K‐edge provides insight into the electronic properties and element‐specific local structural environment. The FT‐EXAFS spectra of the Ag K‐edge for the NC **1–*b***, **1–*d*, 1–*p*** and clicked products (SPAAC and SBL) are shown in Figure 5. Qualitative inspection of the spectra reveals two major peaks, the peak around 1.9 Å is due to the Ag–S bond and the peak around 2.8 Å originates from the Ag–Ag bond. The intensity of the peaks is much lower compared to the peaks found in the Ag foil spectrum, this can be related to the size of the NCs and the Debye‐Weller factor.

Analysis of the Ag Kedge XANES region (Figure 5a,d and g) reveals a higher intensity of the white line (∼2.5 eV) which is related to the increase of a d‐hole population in the AgNCs. The FT EXAFS peaks (Figure 5c,f and i) in the *R*‐space at 1.9 Å and 2.8 Å correspond to the scattering paths of the photoelectron waves from the Ag‐S and Ag–Ag bonds. From a qualitative perspective, it can be established that the Ag–Ag bonds scattering paths remain without significant alteration, which supports that the CS click reaction doesn’t interfere with the core integrity. In addition, modification of the carboxylate ligand through SBL and SPAAC reactions reveals a minor reduction in the Ag–S bond scattering path, ∼0.01 Å for **1–*b*–SPAAC** and ∼0.03 Å for **1–*b*–SBL**. This change suggests that the addition of the phosphine moiety promotes a small contraction in the Ag–S bond, this effect can be related to new ligand interactions such as noncovalent interactions between the aromatic rings and hydrogen bonding of amide groups. For comparison, structural changes have been reported upon solvation of NCs with coordinating solvents of different polarity [38, 39]. Application of XAS for evaluation of cluster integrity after surface modification proves to be a powerful characterization technique that can be complemented with the analysis of S K‐edge for thiolate NCs.

## 3 Conclusion

In this work, two new azide‐functionalized Ag_20_ NCs (**1–*b*** and **1–*d***) were reported and characterized by SCXRD. These clusters have a carbonate template, tert‐butylthiolate ligands, and azide‐functionalized ligands (*p*‐azidomethyl benzoate and 3,5‐diazidobenzoate), adding to the library of azide modified Ag_20_NC capable of CS–click chemistry. Postsynthetically, the clusters were subjected to CS functionalization via click chemistry. In addition to CS**–**SPAAC, the scope of CS‐click reactions on Ag_20_NC was expanded to include CS modification using the nontraceless Staudinger–Bertozzi ligation (CS**–**SBL), representing the first example of SBL–mediated NC functionalization. It was confirmed that the CS**–**SBL reaction can be partially achieved but the steric hindrance on the CS limits the hydrolysis step or promotes the formation of the Staudinger product. Extending the reaction time promotes Ag‐P interactions, which can either alter the stoichiometry of the ligands or generate of a different size of AgNC. Having Ag_20_NC with multiple azide containing ligands (and indeed multiple azides per ligand) allows for dual‐reactivity BCC, involving both CS–SPAAC and CS–SBL on the CS highlighted their potential for sequential reactivity, enabling a dual‐click functionalization approach for the introduction of multiple functional groups. Beyond confirming azide reactivity and successful ligand modification through FT–IR, NMR spectroscopies and XPS, the structural integrity of the cluster core following the click reactions was also examined using XAS. This work offers a new perspective of the CS functionalization through click chemistry, integrating analysis of the chemical changes in the ligands and analysis of the Ag–Ag scattering paths. The combination of characterization techniques employed proved effective in providing valuable structural information, especially when obtaining a suitable crystal is challenging. This approach offers a straightforward strategy for introducing new functionalities while preserving NC size, thereby expanding the potential utility of these systems. A limitation of this approach is that a mixture of click‐adducts can be formed and additional purification steps are needed to obtain pure products.

## 4 Experimental Section

### 4.1 Reagents and Instrumentation

3,5‐diaminobenzoic and sodium nitrite were purchased from Caledon. Acetonitrile (ACN, HPLC grade), dimethylacetamide (DMAc), benzyl bromide, and sodium azide were purchased from Sigma–Aldrich. Silver nitrate, 4‐(bromomethyl)benzoic acid, and anhydrous magnesium sulfate were purchased from Fisher Chemical. Triethylamine was purchased from EDM Millipore. Common laboratory solvents (ethyl acetate and dichloromethane (DCM)) were sourced from Fischer Chemical and Sigma–Aldrich. All reagents and solvents were used as received without further purification.

Silver tert‐butylthiolate was synthesized according to a literature procedure [40]. 1*R*,8*S*,9*s*‐bicyclo [6.1.0]non‐4‐yn‐9‐ylmethanol (*endo*‐BCN) was also prepared according to a literature procedure [41].

IR Spectra were collected on a PerkinElmer Spectrum Two FT‐IR Spectrometer in the 500–4000 cm^−1^ window. ^1^H, ^31^P{^1^H} and ^13^C{^1^H} NMR spectra were collected on a Bruker 400 MHz spectrometer in either CDCl_3_ or DMSO‐*d*_6_. The multiplicity of peaks seen in the ^1^H NMR were assigned as either singlet (s), doublet (d), triplet (t), quartet (q), multiplet (m), or broad (br). UV–Vis absorbance spectra were collected on a CARY 100.

XPS analyses were performed with a Kratos Supra with a monochromatic Al Kα ( 15 mL, 15 kV) as an X‐ray source. The instrument work function was calibrated to give a binding energy of 83.96 eV for metallic gold (4f_7/2_) and the spectrometer dispersion was adjusted to give a binding energy of 932.62 eV for metallic copper (Cu 2p_3/2_). Survey spectra were collected using a pass energy of 160 eV and a step size of 1 eV, while high‐resolution spectra were collected with a pass energy of 20 eV and a step size of 0.1 eV. Charge correction and calibration was done using the main component of the C1s spectrum set to 284.8 eV. CasaXPS Version 2.3.24PR1.0 was used to fit the spectra using Shirley background.

The Ag K‐edge XAS spectra were collected at the Hard X‐ray MicroAnalysis Beamline at the Canadian Light Source. Samples were mounted as powders on Kapton tape and measurements were performed at atmospheric pressure. Athena software [42] was used to process the data with a *k*‐range of 3–11 Å^–1^.

ESI‐MS was performed with an Orbitrap Exploris 120 mass spectrometer with ionization source HESI and ion transfer tube temperature of 250ºC, sheath gas 3.74L/min, aux gas 6.19 L/min, spray voltage 3400 V and 2500 V. Vaporizer temperature 50ºC.

### 4.2 Synthesis of 3,5‐Diazidobenzoic Acid, 3,5‐N_3_‐C_6_H_4_COOH

*3*,*5*‐diazidobenzoic acid was synthesized according to a modified version of a previously published literature procedure [43]. In a 250 mL beaker, 3,5‐diaminobenzoic acid (5.0 g, 33.0 mmol) was dissolved in 40.0 mL of HCl 6.0 M. The solution was cooled on ice and stirred open in the dark. Then a solution of NaNO_2_ (10.0 g, 140.0 mmol in 30.0 mL H_2_O) was added dropwise, resulting in an orange solution. The orange suspension was stirred 20 min, and then a solution of NaN_3_ (6.7 g, 100 mmol in 30.0 mL H_2_O) was added dropwise. The solution bubbled vigorously with each addition and produced a large amount of foam. The solution was allowed to stir an additional 30 min. The solution was filtered, and a red solid was washed with water and dissolved in dichloromethane (DCM). Additional product was extracted from the aqueous mixture using DCM (3 x 70.0 mL). Combined organic phases were dried with anhydrous magnesium sulfate, filtered, and obtained as a red‐brown powder after removing the solvent via rotary evaporation. Crude was purified using a flash column (eluent CHCl_3_:MeOH 9:1) to obtain the product as a beige powder. Yield = 5.83 g (>87%). ^1^H NMR (DMSO‐*d*_6_, 400 MHz): δ (ppm) 13.53 (b, 1H, OH), 7.37 (d, ^4^ *J*_*HH*_ = 2.1 Hz, 2H, Ar H), 7.08 (t, ^4^ *J*_*HH*_ = 2.1 Hz, 1H, Ar H). ^13^C{^1^H} NMR (DMSO‐*d*_6_, 400 MHz): δ (ppm) 165.74, 141.62, 130.73, 116.01, 113.96. FT‐IR (ATR): azide stretching 2119 (s) cm^−1^

### 4.3 Synthesis of *p*‐Azidomethyl Benzoic Acid, *p*‐(N_3_‐CH_2_)‐C_6_H_4_COOH

*p*‐azidomethylbenzoic acid was synthesized according to a modified version of a previously published literature procedure [24]. To a suspension of 4‐(bromomethyl)benzoic acid (1000 mg, 4.65 mmol) in 30.0 mL ACN, NaN_3_ (400 mg, 6.15 mmol) was added. The mixture was heated at 85°C and refluxed for 14 h. The solvent was removed by rotary evaporation. A mixture of DCM and water (1:1) was added to remove the unreacted sodium azide through extraction. The aqueous layer was acidified to pH 2 with HCl 1 M and extracted with DCM to collect the product. The collected organic phases were dried with anhydrous magnesium sulfate and filtered. The solvent was evaporated, and a white solid was collected. Yield = 0.659 g (80.1%) ^1^H NMR (DMSO‐*d*_6_, 400 MHz): δ (ppm) 13.03 (s, 1H, OH), 7.96 (d, ^3^ *J*_*HH*_ = 8.4 Hz, 2H, Ar H), 7.48 (d, ^3^ *J*_*HH*_ = 8.4 Hz, 2H, Ar H), 4.57 (s, 2H, CH_2_).

### 4.4 Synthesis of [(CO_3_)@Ag_20_(S^*t*^Bu)_10_(3,5‐N_3_‐C_6_H_4_COO)_8_(DMAc)_4_] (1‐*d*)

In a 100 mL beaker, AgNO_3_ (50.0 mg, 0.294 mmol) and [AgS^*t*^Bu]_*n*_ (57.4 mg, 0.291 mmol) were dissolved in a mixture of DMAc:ACN (1:1). The mixture was sonicated until the reagents were fully dissolved and a colorless solution was obtained. *3*,*5*‐diazidobenzoic acid (48.2 mg, 0.236 mmol) was added as a powder. Triethylamine 10 μL was then added to the reaction mixture. The mixture turned slightly yellow, and it was allowed to slowly evaporate for 2 days, yielding colorless block crystals. The crystals were washed with ACN (3 x 2 mL). Yield = 127.0 mg (84.9%)

^1^H NMR (CDCl_3_, 600 MHz): δ (ppm) 7.59 (d, ^4^ *J*_*HH*_ = 2.1 Hz, 16H, Ar H), 6.72 (t, ^4^ *J*_*HH*_ = 2.2 Hz, 8H, Ar H), 3.02 (s, 12H, CH_3_), 2.95 (s, 12H, CH_3_), 2.09 (s, 12H, CH_3_), 1.73 (s, 90H, CH_3_).

FT‐IR (ATR): azide stretching 2112 (s) cm^−1^.

### 4.5 Synthesis of [(CO_3_)@Ag_20_(S^*t*^Bu)_10_(*p*‐(N_3_‐CH_2_)C_6_H_4_COO)_6_(NO_3_)_2_(DMAc)_4_] (1‐*b*)

In a 100 mL beaker, AgNO_3_ (50.0 mg, 0.294 mmol) and [AgS^*t*^Bu]_*n*_ (57.5 mg, 0.291 mmol) were dissolved in a mixture of DMAc:ACN (1:1). The mixture was sonicated until the reagents were fully dissolved and a colorless solution was obtained. p‐(azidomethyl)benzoic acid (41.8 mg, 0.236 mmol) was added as a powder. Triethylamine 10 μL was then added to the reaction mixture. The mixture turned slightly yellow, and it was allowed to slowly evaporate for 2 days, yielding colorless block crystals. The crystals were washed with ACN (3 x 2 mL). Yield = 114.0 mg (83.5%)

^1^H NMR (CDCl_3_, 400 MHz): δ (ppm) 8.20 (d, ^3^ *J*_*HH*_ = 8.2 Hz, 12H, Ar H), 7.38 (d, ^3^ *J*_*HH*_ = 7.9 Hz, 12H, Ar H), 4.42 (s, 12H, CH_2_), 2.94 (s, 12H, CH_3_), 2.09 (s, 12H, CH_3_), 1.67 (s, 90H, CH_3_). FT‐IR (ATR): azide stretching 2096 cm^−1^.

### 4.6 Synthesis of [(CO_3_)@Ag_20_(S^*t*^Bu)_10_(*p*‐N_3_‐C_6_H_4_COO)_6_(DMAc)_4_] (1‐*p*)

[CO_3_@Ag_20_(4‐N_3_‐C6H4COO)_8_(S^t^Bu)_10_] was synthesized according to a previously established procedure, with minor revisions [16]. [AgS^t^Bu]_*n*_ (246 mg, 1.25 mmol) and AgNO_3_ (200 mg, 1.25 mmol) were dissolved in DMAc (20 mL) and MeCN (20 mL), in a 200 mL beaker. The solution was sonicated until clear. To the solution, 4‐azidobenzoic acid (165 mg, 1.00 mmol) was added, followed by the addition of Et_3_N (50 μL). The beaker was wrapped in aluminum foil and left to evaporate slowly. Over 3 days, small, plate‐like crystals formed. The supernatant was removed, the precipitate was transferred to a 15 mL falcon tube, and Et_2_O (15 mL) was added, and centrifuged (4000 rpm, 5 min). The supernatant was removed, and to the remaining precipitate was added CHCl_3_ (15 mL) was added before centrifugation again (4000 rpm, 5 min). The supernatant was separated and concentrated, providing the product as an off‐white solid (500 mg, 88%). ^1^H NMR (CDCl_3_, 400 MHz) δ 8.15 (d, ^3^ *J*_HH_ = 8.5 Hz, 16H, Ar H), 7.06 (d, ^3^ *J*_HH_ = 8.5 Hz, 16H, Ar H), 1.67 (br, 90H, CH_3_). FT‐IR (ATR): 2115, 2076, 1601, 1589, 1456, 1372, 1284, 1152, 775 cm^–1^.

### 4.7 Synthesis of [(CO_3_)@Ag_20_(S^*t*^Bu)_10_(3,5‐(HOC_10_H_13_N_3_)_2_‐C_6_H_3_COO)_8_(DMAc)_4_] (1‐*d*‐SPAAC)

In a 25 mL round bottom flask, BCN_endo_ (18.0 mg, 0.119 mmol) and 1‐*d* (30.0 mg, 0.006 mmol) were combined and dissolved in 15 mL of DCM. A clear, colorless solution was obtained and stirred at room temperature. The mixture was stirred overnight, covered with foil at room temperature, and a beige precipitate was collected through centrifugation the next day. The white precipitate was washed three times with 10 mL of DCM. Yield = 14.2 mg (38.3%)

^1^H NMR (DMSO‐*d*_6_, 400 MHz): δ (ppm) 8.09 (bs, 16H, Ar H), 7.73 (bs, 8H, Ar H), 4.33 – 4.25 (m, 32H, CH_2_), 3.48 – 3.43 (m, 64H, CH_2_), 2.83 (s, 8H, CH), 2.68 – 2.65 (m, 8H, CH), 1.64 – 1.53 (br, 90H, CH_3_), 1.45 (s, 16H, OH), 1.23 (s, 16H, CH), 0.95 (br, 32H).

### 4.8 Synthesis of [(CO_3_)@Ag_20_(S^*t*^Bu)_10_(*p*‐HOC_10_H_13_N_3_‐CH_2_C_6_H_4_COO)_6_(DMAc)_4_] (1‐*b*‐SPAAC)

In a 25 mL round botton flask, BCN_endo_ (6.7 mg, 0.041 mmol) and 1‐*b* (20.0 mg, 0.004 mmol) were combined and dissolved in 15 mL of DCM. The reaction mixture was stirred to give a clear, pale‐yellow solution. After 15 min, the mixture became cloudy. The reaction mixture was stirred at room temperature covered with foil overnight. The next day, the beige precipitate was isolated through centrifugation and washed with DCM (3x10 mL), obtaining a beige solid. Yield = 7.3 mg (31.3%)

^1^H NMR (DMSO‐*d*_6_, 400 MHz): δ (ppm) 7.95 (bs, 12H, Ar H), 7.13 (bs, 12H, Ar H), 5.61 (bs, 12H, Ar H), 4.34 – 4.23 (bs, 12H, CH_2_), 3.42 (bs, 12H, CH_2_), 3.09 – 2.69 (m, 24H, CH), 2.62 (bs, 6H, OH), 2.08 – 1.83 (m, 12H, CH), 1.43 (bs, 90H, CH_3_), 0.91 (s, 7H, CH), 0.68 (s, 6H, CH).

### 4.9 Synthesis of 2‐Diphenylphosphanyl Benzoic Acid Methyl Ester (C_20_H_17_O_2_P)

2‐Diphenylphosphanyl Benzoic Acid Methyl Ester was synthesized according to a previous procedure [26]. In a round bottom flask, 80 mL of HCl 4 M and methyl‐2‐aminobenzoate (2.33 g, 15.0 mmol) were dissolved at 0°C. Then an aqueous solution of sodium nitrite (1.17 g, 16.0 mmol) was added dropwise to the reaction flask and stirred for 20 min. An aqueous solution of potassium iodide (13.0 g in 100 mL of water) was added dropwise; the reaction mixture turns dark brown, and it is stirred at room temperature for an additional 4 h. A saturated sodium sulfite solution (70 mL) was added to quench the reaction until it turned yellow, and the crude was extracted with dichloromethane (3 x 20 mL). The organic phases were combined, dried with anhydrous MgSO_4_ and concentrated. The product (methyl‐2‐iodobenzoate) was obtained after flash column purification using DCM:Hexanes (3:1) as eluent. Yield = 1.71 g (42%). ^1^H NMR (400 MHz, Chloroform‐*d*) δ (ppm) 8.00 (dd, ^3^ *J*_*HH*_ = 7.9, ^4^ *J*_*HH*_ = 1.2 Hz, 1H, Ar H), 7.80 (dd, ^3^ *J*_*HH*_ = 7.8, ^4^ *J*_*HH*_ = 1.7 Hz, 1H, Ar H), 7.40 (td, ^3^ *J*_*HH*_ = 7.6, ^4^ *J*_*HH*_ = 1.2 Hz, 1H, Ar H), 7.15 (td, ^3^ *J*_*HH*_ = 7.7, ^4^ *J*_*HH*_ = 1.7 Hz, 1H, Ar H), 3.94 (s, 3H, CH_3_).

The methyl‐2‐iodobenzoate obtained in the previous step and palladium acetate (0.15 g, 0.665 mmol) were dissolved in 15.0 mL of dry ACN. Anhydrous triethylamine (2.70 mL, 19.4 mmol) and dipheylphosphine (1.35 mL, 7.78 mmol) were then added under argon. The reaction mixture was heated to reflux and stirred overnight under an argon atmosphere. The reaction mixture was then cooled down to room temperature and poured into a flask containing HCl (100 mL, 1 M). The crude was extracted with DCM (3 × 30 mL), the combined organic phases were dried with anhydrous MgSO_4_, filtered and concentrated. The crude residue was then purified by flash column (ethyl acetate: hexanes 1:4) to yield an off‐white product, which was recrystallized from methanol to provide 2‐diphenylphosphanyl benzoic acid methyl ester as an off‐white solid. Yield = 1.14 g (55%). ^1^H NMR (400 MHz, Chloroform‐*d*) δ (ppm) 8.05 (m, 1H, Ar H), 7.40 – 7.29 (m, 12H, Ar H), 6.93 (m, 1H, Ar H), 3.74 (s, 3H, CH_3_). ^31^P{^1^H} NMR (162 MHz, CDCl_3_) δ (ppm) – 4.28.

### 4.10 Staudinger–Bertozzi Adduct: [(CO_3_)@Ag_20_(S^*t*^Bu)_10_(3,5‐(POC_19_H_15_ON)_2_‐C_6_H_3_COO)_8_(DMAc)_4_] (1‐*d*‐SBL)

In a 25 mL round bottom flask equipped with a stir bar, **1‐*d*** (91.3 mg, 0.018 mmol) was dissolved in 10 mL of DCM. Then methyl 2‐(diphenylphosphino)benzoate (115.0 mg, 0.359 mmol) was dissolved in 5 mL DCM and added to the reaction flask. The reaction mixture was stirred at room temperature at 600 rpm for 1 h, then milliQ water (100 μL) was added to the mixture. The mixture was stirred for an additional 30 hr. The reaction mixture was then partially concentrated, and the crude concentrate was precipitated from hexanes (40 mL) in a Falcon tube and centrifuged (4500 rpm, 5 min). Two more times, the supernatant was decanted off, the crude residue was redissolved in minimum DCM, and then reprecipiated from hexanes and centrifuged (4500 rpm, 5 min). Following the last centrifugation, the supernatant was decanted, and the precipitate was isolated, dried under vacuum to yield the product **1‐*d*‐SBL** as a white solid. Yield = 83.7 g (49%). ^1^H NMR (400 MHz, Chloroform‐*d*): δ (ppm) 10.59 (s, 1H, NH), 8.16 (d, ^3^ *J*_HH_ = 7.5 Hz, 8H, Ar H), 7.91 – 7.89 (m, 1H, Ar H), 7.85 – 7.83 (m, 4H, Ar H), 7.75 – 7.71 (m, 14H, Ar H), 7.68 – 7.59 (m, 14H, Ar H), 7.54 – 7.49 (m, 20H, Ar H), 7.45 – 7.41 (m, 59H, Ar H), 7.36 – 7.33 (m, 29H, Ar H), 7.13 (s, 3H, Ar H), 6.94 (d, ^3^ *J*_HH_ = 7.5 Hz, 8H, Ar H), 6.29 (s, 3H, Ar H), 3.64 (s, 12H), 3.48 (s, 1H, NH), 3.29 (s, 9H, NH), 1.58 (s, 90H, CH_3_). ^31^P{^1^H} NMR (162 MHz, CDCl_3_) δ 30.74.

### 4.11 Staudinger–Bertozzi Adduct: [(CO_3_)@Ag_20_(S^*t*^Bu)_10_(*p*‐POC_19_H_15_ON‐CH_2_C_6_H_4_COO)_6_(DMAc)_4_] (1‐*b*‐SBL)

In a 25 mL round bottom flask equipped with a stir bar, **1‐*b*** (100.0 mg, 0.021 mmol) was dissolved in 10 mL of DCM. Then methyl 2‐(diphenylphosphino)benzoate (65.8 mg, 0.205 mmol) was dissolved in 5 mL DCM and added to the reaction flask. The reaction mixture was stirred at room temperature at 600 rpm; after 1 h 100 μL of miliQ water was added to the mixture. The mixture was stirred for an additional 30 hr. The reaction mixture was then partially concentrated, and the crude concentrate was precipitated from hexanes (40 mL) in a Falcon tube and centrifuged (4500 rpm, 5 min). Two more times, the supernatant was decanted off, the crude residue was redissolved in minimum DCM, and then reprecipiated from hexanes and centrifuged (4500 rpm, 5 min). Following the last centrifugation, the supernatant was decanted, and the precipitate was isolated and dried under vacuum, to yield the product **1‐*b*‐SBL** as a white solid. Yield = 77.2 mg (60%). ^1^H NMR (400 MHz, Chloroform‐*d*): δ (ppm) 9.13 (s, 1H, NH), 8.18 (d, ^3^ *J*_HH_ = 7.7 Hz, 12H, Ar H), 8.14 (d, ^3^ *J*_HH_ = 7.8 Hz, 6H, Ar H), 8.07 – 7.99 (m, 4H, Ar H), 7.64 – 7.63 (m, 12H, Ar H), 7.56 – 7.55 (m, 6H, Ar H), 7.46 – 7.43 (m, 55H, Ar H), 7.21 (d, *J* = 7.7 Hz, 6H, Ar H), 7.14 – 7.04 (m, 2H, Ar H), 6.96 (d, ^3^ *J*_HH_ = 7.7 Hz, 12H, Ar H), 4.38 (s, 8H, CH_2_), 4.15 (d, ^3^ *J*_HH_ = 5.7 Hz, 6H, CH_2_), 3.69 (s, 2H, NH), 1.65 – 1.61 (m, 90H, CH_3_). ^31^P{^1^H} NMR (162 MHz, CDCl_3_) δ 30.68.

### 4.12 Staudinger–Bertozzi Adduct: [CO_3_@Ag_20_(*p*‐POC_19_H_15_ON‐C_6_H_4_COO)_8_(StBu)_10_] (1‐*p*‐SBL)

[CO_3_@Ag_20_(4‐N_3_‐C_6_H_4_COO)_8_(StBu)_10_] (60 mg, 0.01 mmol) was dissolved in CH_2_Cl_2_ (2 mL) in a vial, followed by the addition of methyl 2‐(diphenylphosphino)benzoate (60 mg, 0.18 mmol). One drop of water was added to the vial, and the vial was left overnight, stirring and wrapped in aluminum foil. The solution was then transferred to a 45 mL falcon tube and diluted to 45 mL using hexanes (43 mL). The reaction mixture was centrifuged (4000 rpm, 5 min), generating a white precipitate. The supernatant was removed, and the precipitate was redissolved in minimum CH_2_Cl_2_. Twice more, the solution was diluted to 45 mL using hexanes (43 mL) and centrifuged (4000 rpm, 5 min), removing the supernatant each time before redissolution in CH_2_Cl_2_. After the final centrifugation, the supernatant was removed, and the precipitate was dried in vacuo (90 mg, 66%). Assignments of proton signals to unreacted azide, to azaylide intermediate and to the Staudinger–Bertozzi phosphine oxide adduct were made based on coupling constants and comparing experiments where different ratios of azaylide to phosphine were observed. ^1^H NMR (DMSO‐d_6_, 400 MHz) δ 8.18 (d, *J* = 7.4 Hz, 5H, Ar H), 8.15 (d, ^3^ *J*_HH_ = 8.5 Hz, 5H, Ar H), 7.96 (d, ^3^ *J*_HH_ = 8.5 Hz, 5H, Ar H), 7.82–7.30 (m, 85H, Ar H), 7.01 (d, ^3^ *J*_HH_ = 8.5 Hz, 6H, Ar H), 6.98 (d, ^3^ *J*_HH_ = 7.4 Hz, 5H, Ar H), 6.66 (d, ^3^ *J*_HH_ = 7.4 Hz, 5H, Ar H), 3.67 (br, 16H, NH), 1.68 (br, 90H, CH_3_). ^31^P{^1^H} NMR (DMSO‐d_6_, 162 MHz) δ 30.73

CCDC 2 484 704 and 2 484 705 contains the supplementary crystallographic data for the structures **1‐*b*** and **1‐*d***, respectively presented in this paper. These data can be obtained free of charge from The Cambridge Crystallographic Data Centre via www.ccdc.cam.ac.uk

## Supporting Information

Additional supporting information can be found online in the Supporting Information section.

## Funding

Canada Foundation for Innovation (CFI‐IF #35961), Ontario Research Fund and NSERC‐DG Canada.

## Conflicts of Interest

The authors declare no conflicts of interest.

## Supporting information

Supplementary Material

## Data Availability

CCDC 2484704 and 2484705 contains the supplementary crystallographic data for the structures **1‐*b*** and **1‐*d***, respectively presented in this paper.
