# Supplementary material for: Dual Cluster‐Surface Click Chemistries on Silver Nanoclusters
Source: Small Sci. 2026 Apr 27;6(5):e70289. doi: 10.1002/smsc.70289 (PMC13120733; doi:10.1002/smsc.70289)
Supplement: Supplementary file 1 — Supplementary Material [file SMSC-6-e70289-s001.pdf]

Supporting Information

Dual Cluster-Surface Click Chemistries on Silver Nanoclusters

Carolina Vega Verduga, *Jack E. Bowman, Jeffrey D. Henderson^, Paul D. Boyle, John F. Corringan*[#], *Mark S. Workentin**

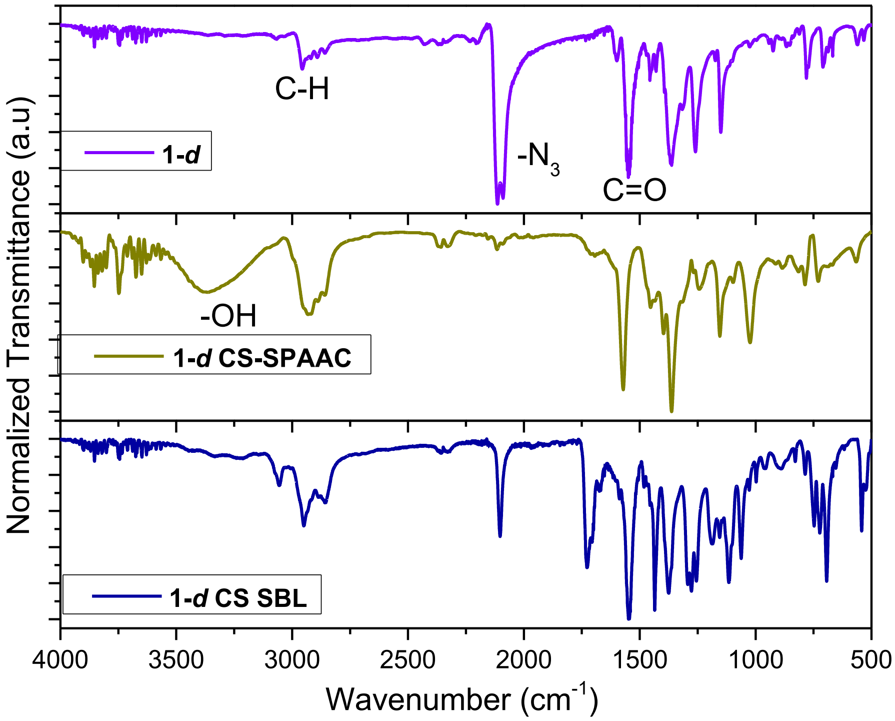

Figure S1 Stacked FTIR spectra of **1** ***d*** and its cluster surface reaction CS –SPAAC and CS –SBL

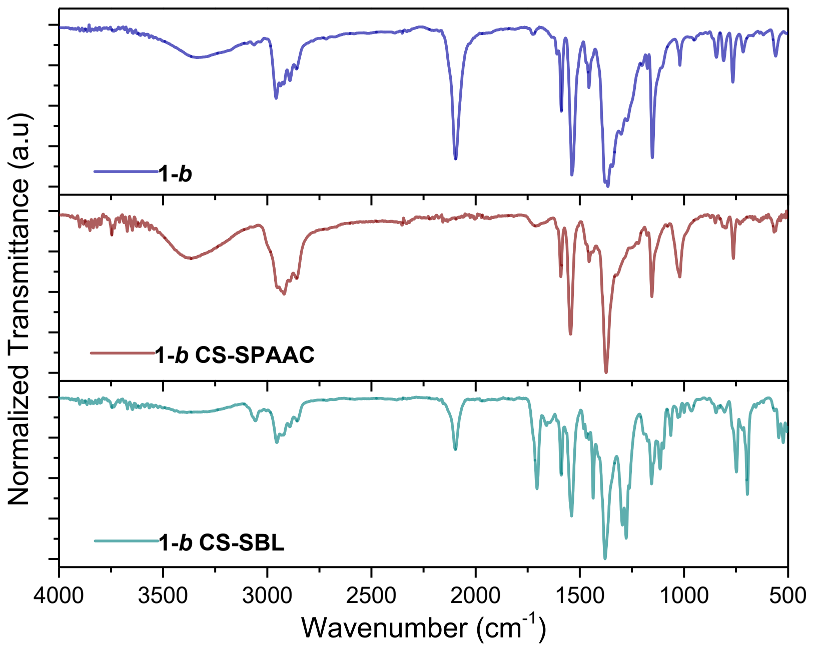

Figure S2 Stacked FTIR spectra of **1** *b* and its cluster surface reaction CS –SPAAC and CS –SBL

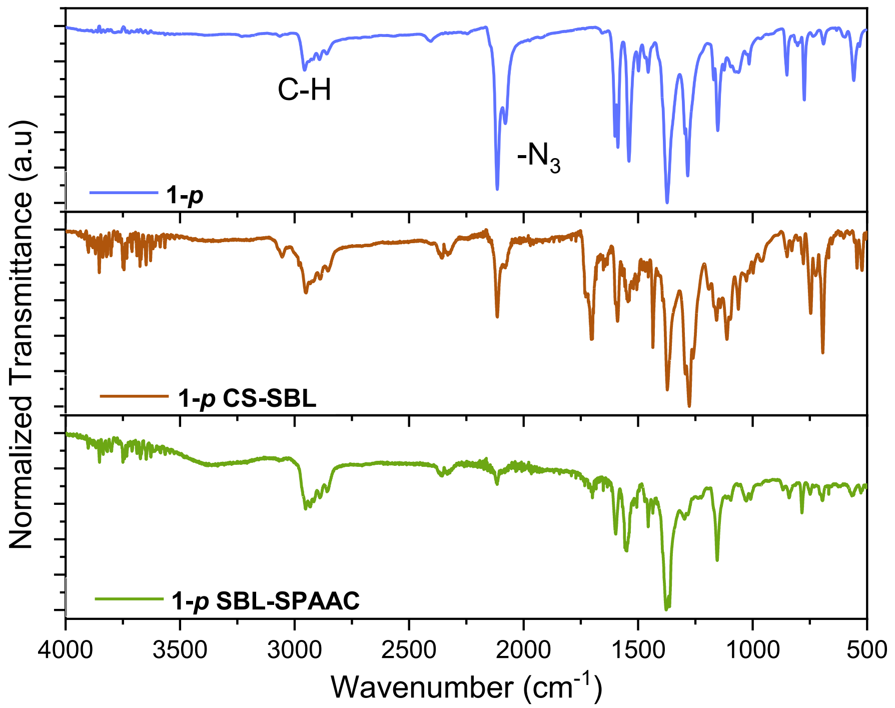

Figure S3 FT –IR spectra of a) free **1** *p* nanocluster b) model phosphine against the c) and d) click adduct **1** *p* **SBL** at different reaction times.

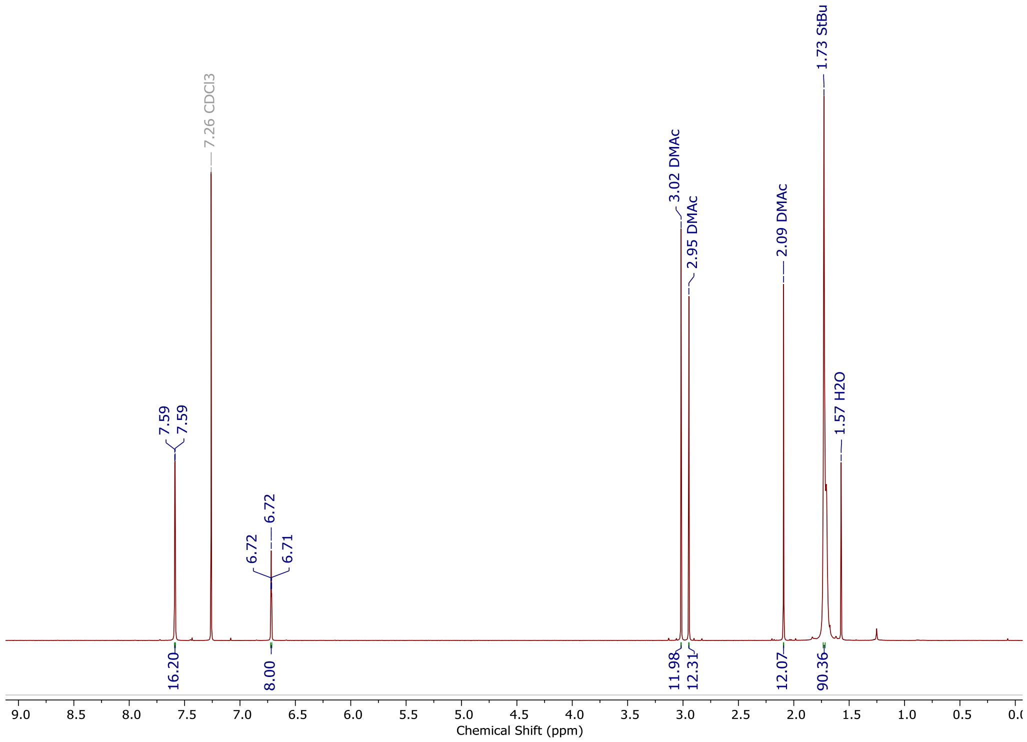

Figure S4 ^{1}H NMR spectrum of [$(CO_3)@Ag_{20}(S^tBu)_{10}(3,5\text{-}(N_3)_2\text{-}C_6H_4COO)_8(DMAc)_4$] (**1** ***d***) in $CDCl_3$.

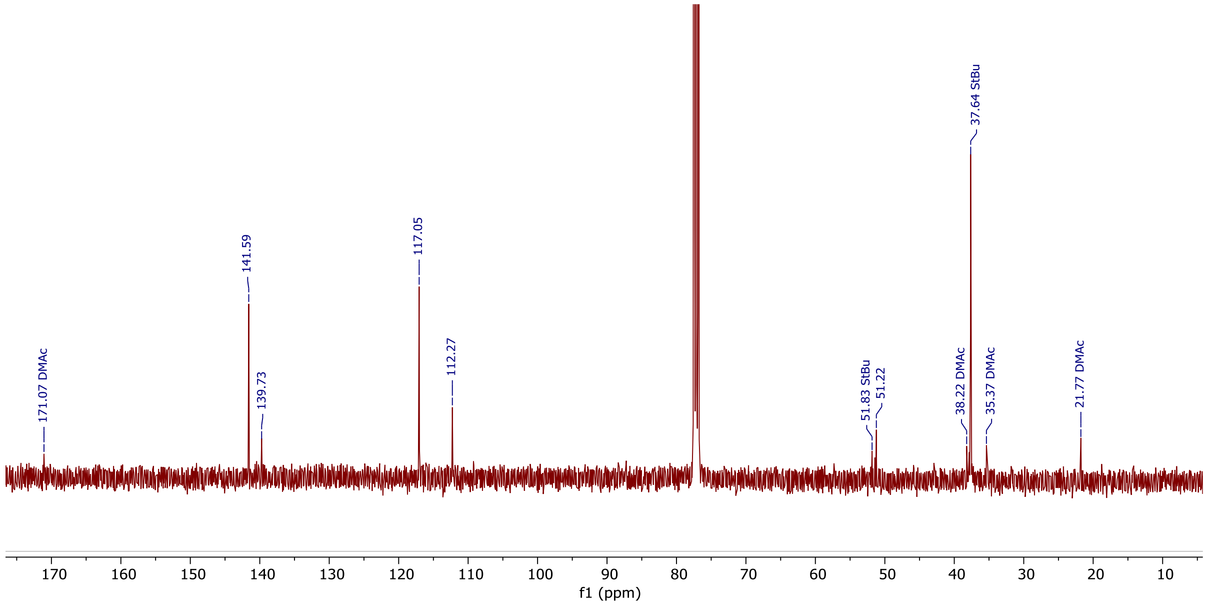

Figure S5 $^{13}C\{^{1}H\}$ NMR spectrum of [$(CO_3)@Ag_{20}(S^tBu)_{10}(3,5\text{-}(N_3)_2\text{-}C_6H_4COO)_8(DMAc)_4$] (**1** ***d***) in $CDCl_3$.

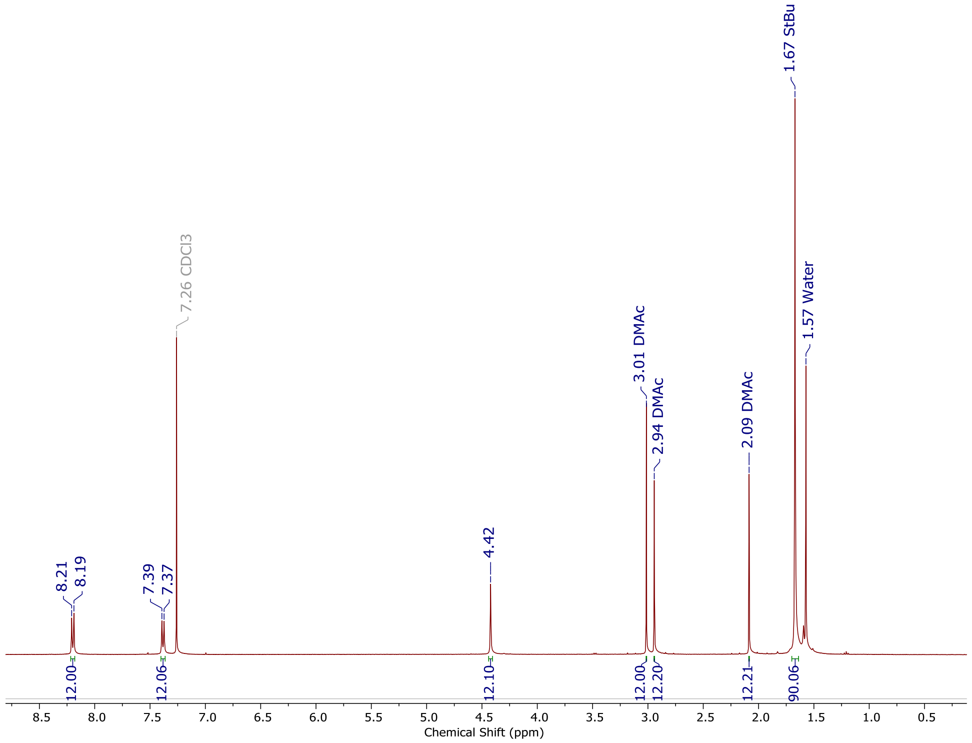

Figure S6 ^{1}H NMR spectrum of $[(CO_3)@Ag_{20}(S^tBu)_{10}(p\text{-}(N_3\text{-}CH_2)C_6H_4COO)_6(DMAc)_4]$ (**1** ***b***) in $CDCl_3$

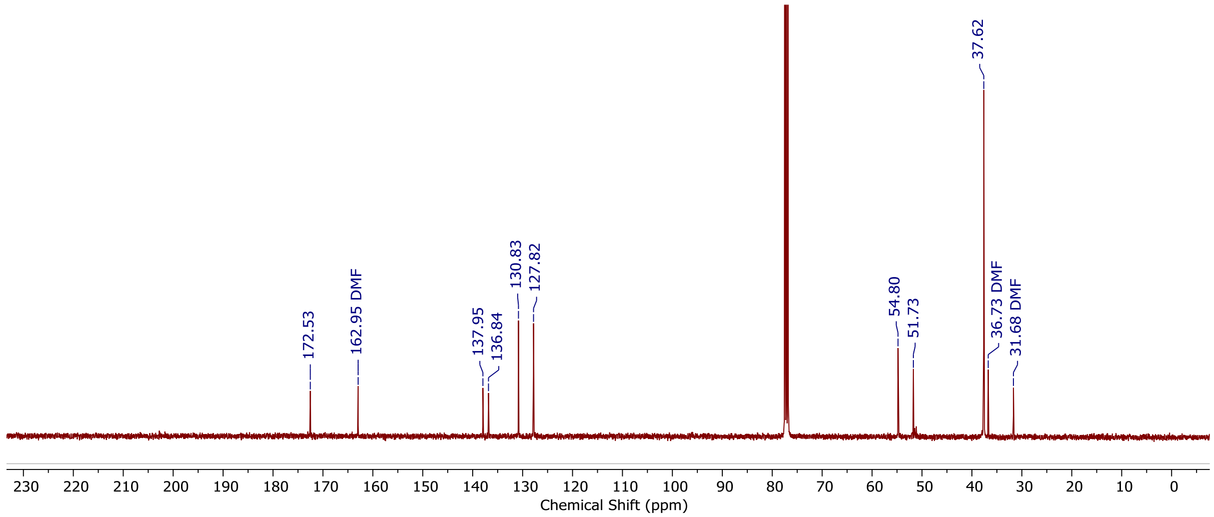

Figure S7 $^{13}C\{^{1}H\}$ NMR spectrum of $[(CO_3)@Ag_{20}(S^tBu)_{10}(p\text{-}(N_3\text{-}CH_2)C_6H_4COO)_6(DMF)_4]$ (**1** ***b***) in $CDCl_3$

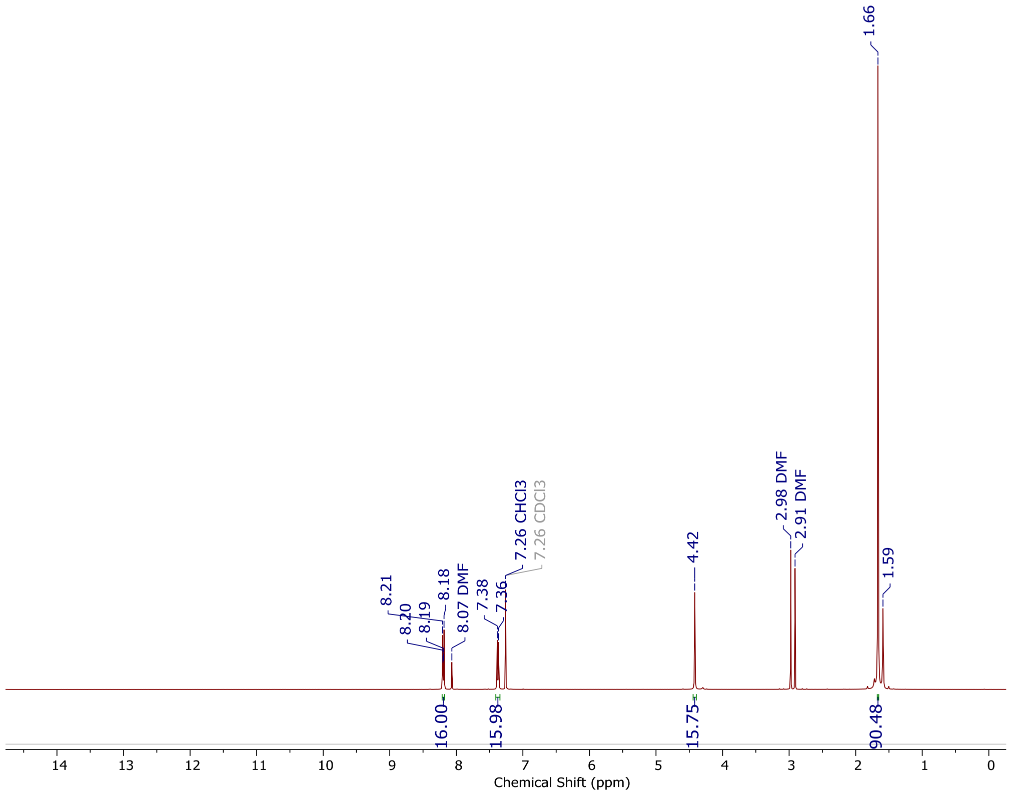

Figure S8 ^{1}H NMR spectrum of $[(CO_3)@Ag_{20}(S^tBu)_{10}(p\text{-}(N_3\text{-}CH_2)C_6H_4COO)_8(DMF)_4]$ (**1** ***b***) in $CDCl_3$

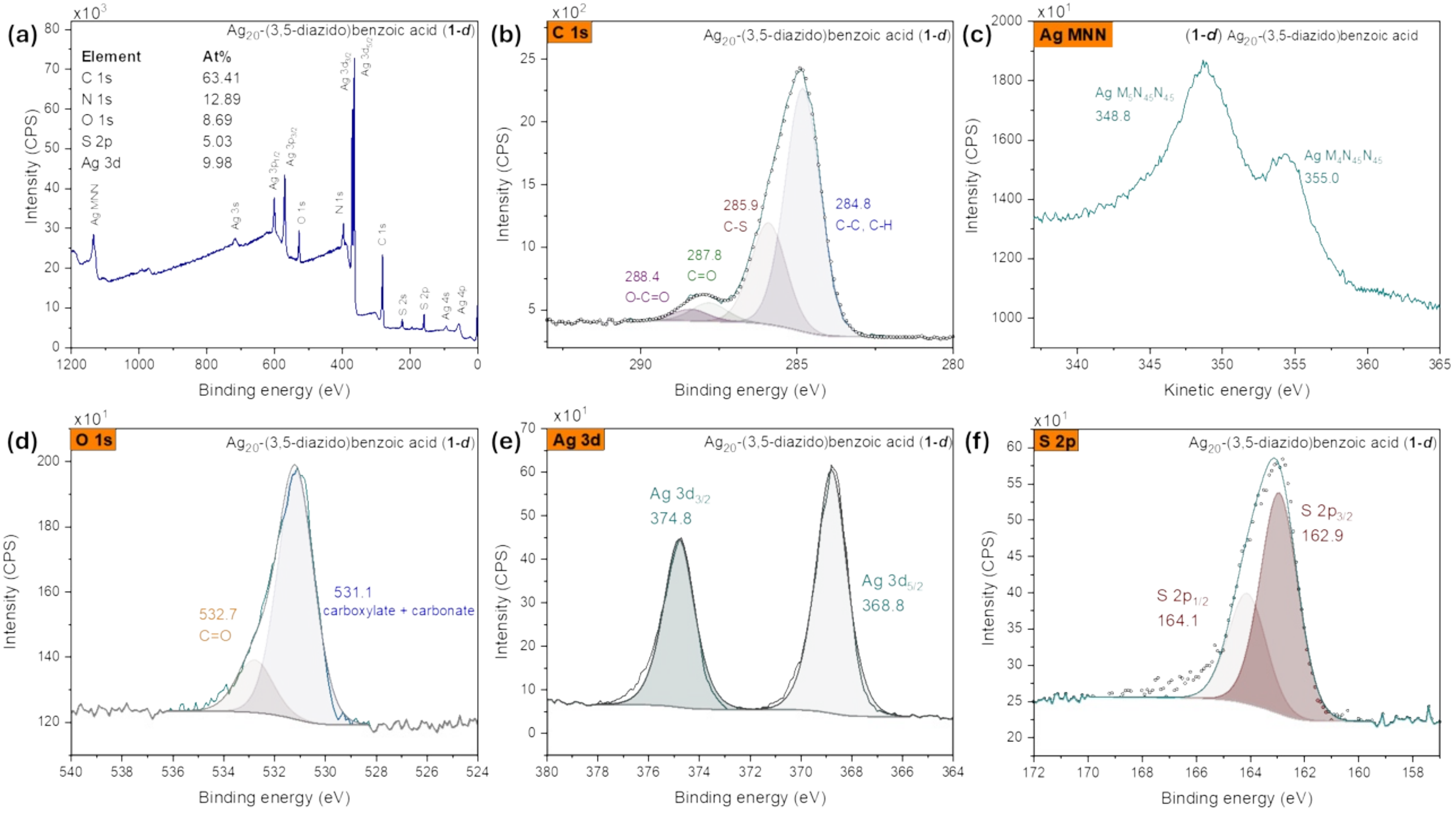

Figure S9 High resolution XPS spectra of **1** ***d*** (a) survey spectrum (b) C 1s (c) Ag MNN (d) O 1s (e) Ag 3d (f) S 2p. C 1s was fitted with C-C 284.8 eV, C-S 285.9 eV, C=O 288.8 eV, and carbonate 288.4 eV. Ag 3d was fitted with two spin-orbit components Ag $3d_{3/2}$ and Ag $3d_{5/2}$. S 2p was fitted considering S $2p_{1/2}$ and S $2p_{3/2}$ spin-orbit components with fixed doublet separation of 1.18eV and constrained area ratio; S $2p_{3/2}$ Ag-S 162.9 eV. O 1s was fitted with two components: 531.1 eV corresponding to carboxylate and carbonate species and 532.7 eV assigned to C=O.

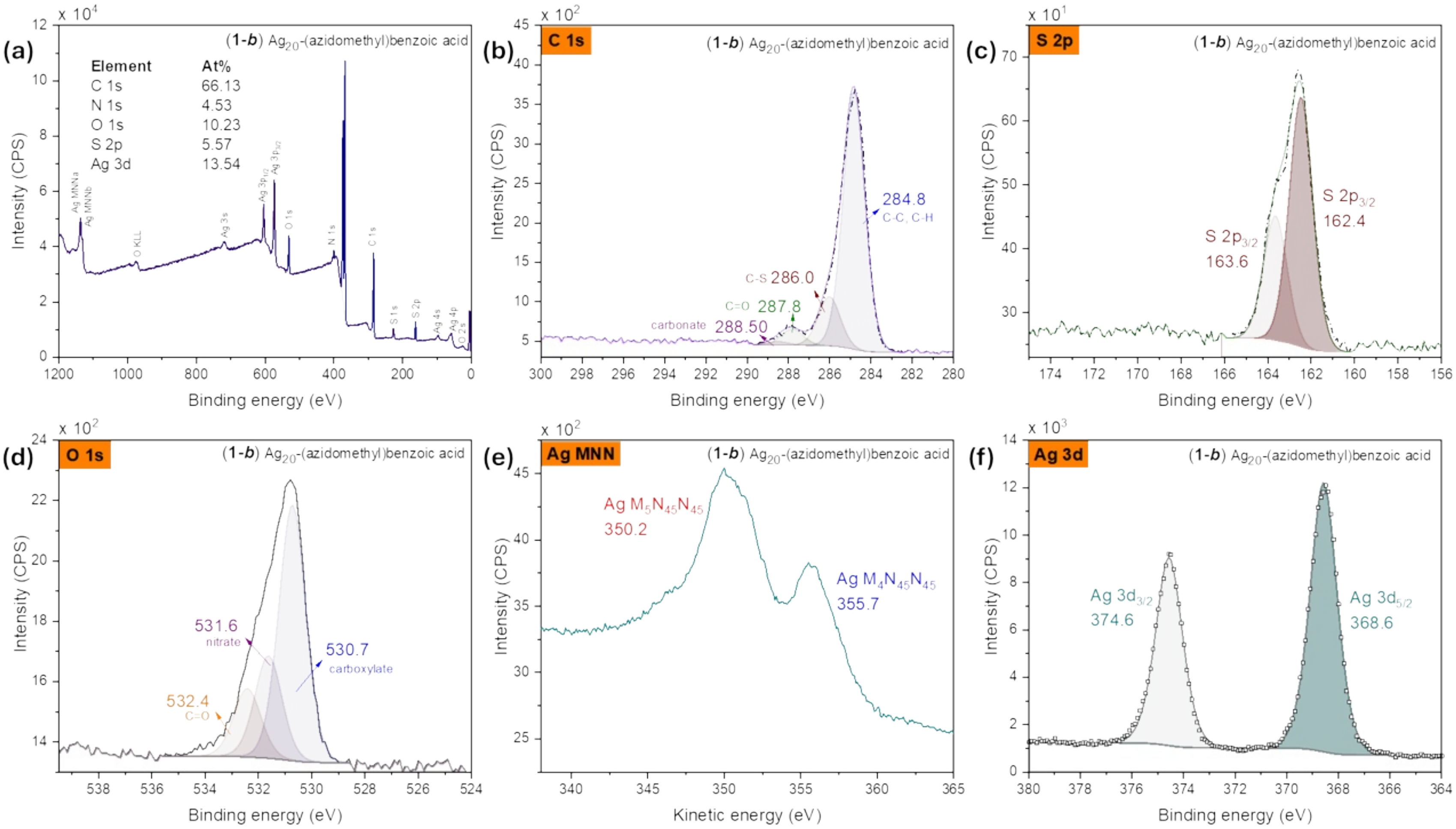

Figure S10 High resolution XPS spectra of **1** ***b*** (a) survey spectrum (b) C 1s (c) S 2p (d) O 1s (e) Ag MNN (f) Ag 3d. C 1s was fitted with C-C 284.8 eV, C-S 286.0 eV, C=O 287.8 eV, and carbonate 288.5 eV. Ag 3d was fitted with two spin-orbit components Ag $3d_{3/2}$ and Ag $3d_{5/2}$. S 2p was fitted considering S $2p_{1/2}$ and S $2p_{3/2}$ spin-orbit components with fixed doublet separation of 1.18eV and constrained area ratio; S $2p_{3/2}$ Ag-S 162.4 eV. O 1s was fitted with three components: 530.7 corresponding to carboxylate, 531.6 eV carbonate + nitrate species and 532.7 eV assigned to C=O.

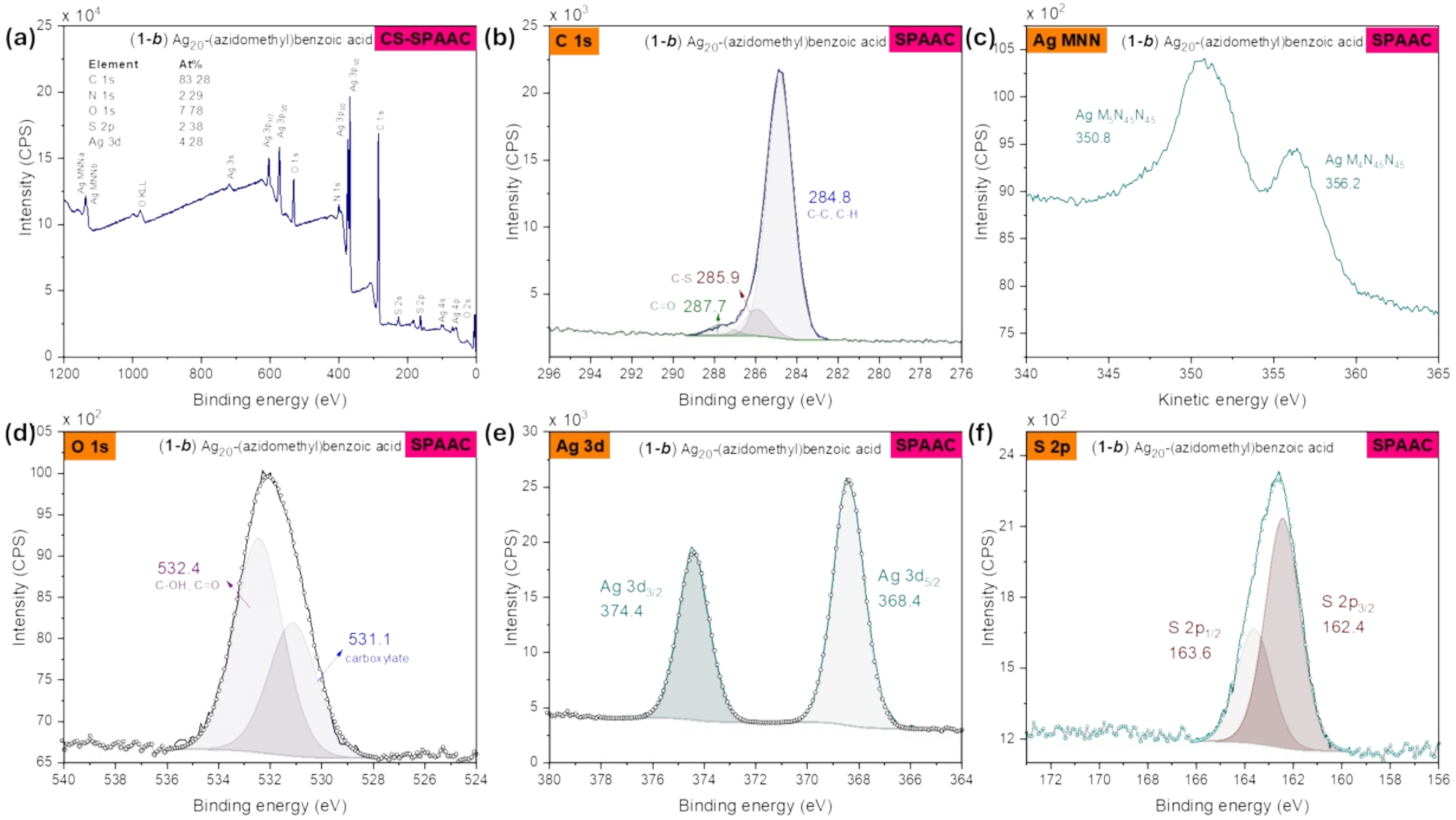

Figure S11 High resolution XPS spectra of **1** ***b*** + BCN (**1** ***b*** **SPAAC**) (a) survey spectrum (b) C 1s (c) Ag MNN (d) O 1s (e) Ag 3d (f) S 2p. C 1s was fitted with C-C 284.8 eV, C-S 285.9 eV, and C=O 287.7 eV. Ag 3d was fitted with two spin-orbit components Ag $3d_{3/2}$ and Ag $3d_{5/2}$. S 2p was fitted considering S $2p_{1/2}$ and S $2p_{3/2}$ spin-orbit components with fixed doublet separation of 1.18eV and constrained area ratio; S $2p_{3/2}$ Ag-S 162.4 eV. O 1s was fitted with two components: 531.1 corresponding to carboxylate, and 532.4 eV carbonate + OH + C=O

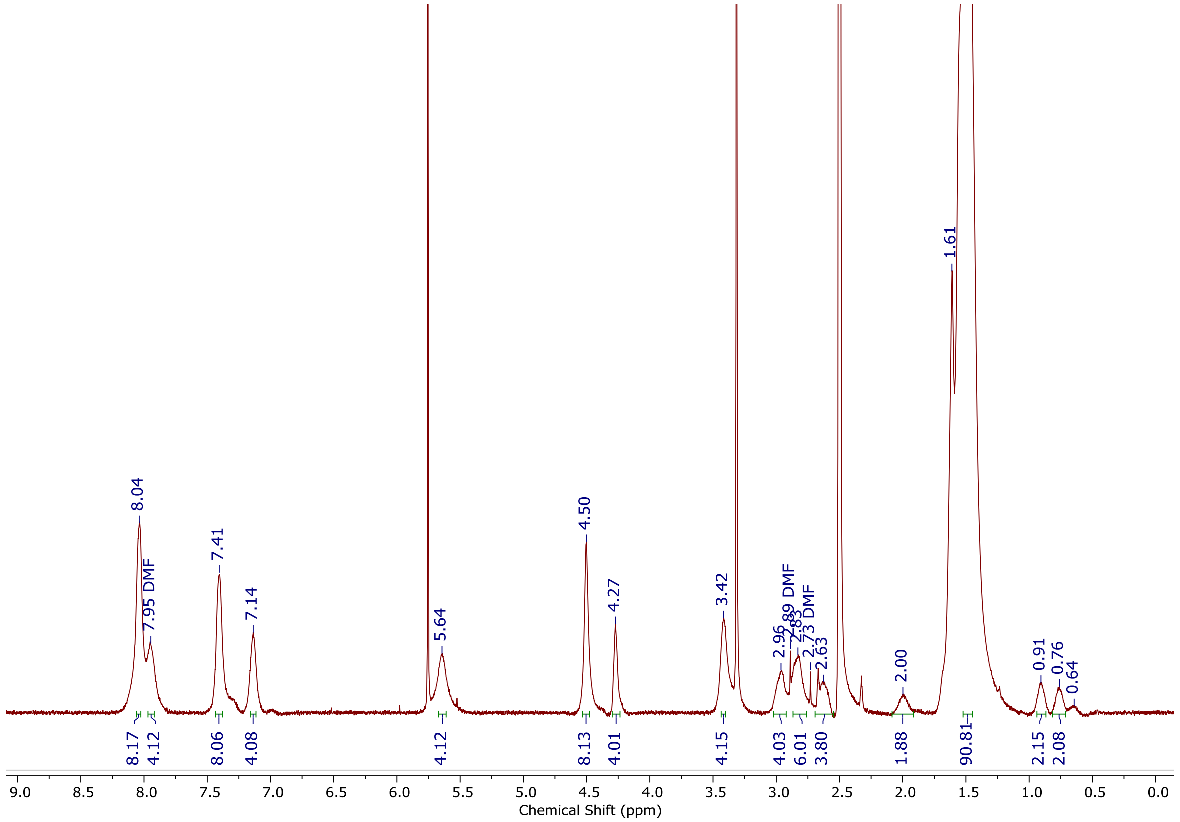

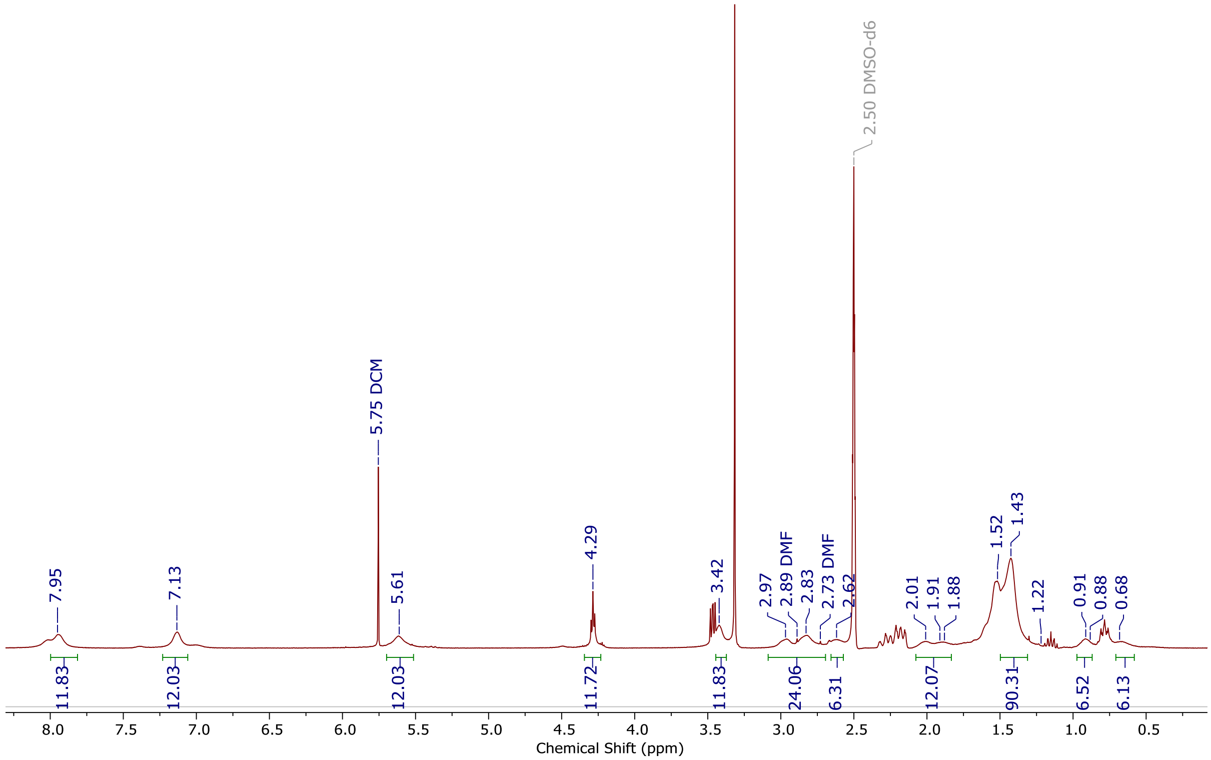

Figure S12 ^{1}H NMR spectrum of $[(CO_3)@Ag_{20}(S^tBu)_{10}(p\text{- }HOC_{10}H_{13}N_3\text{-}CH_2C_6H_4COO)_6(DMF)_4]$ (**1** ***b*** + BCN) in DMSO-d_6. Top spectrum partial reaction.

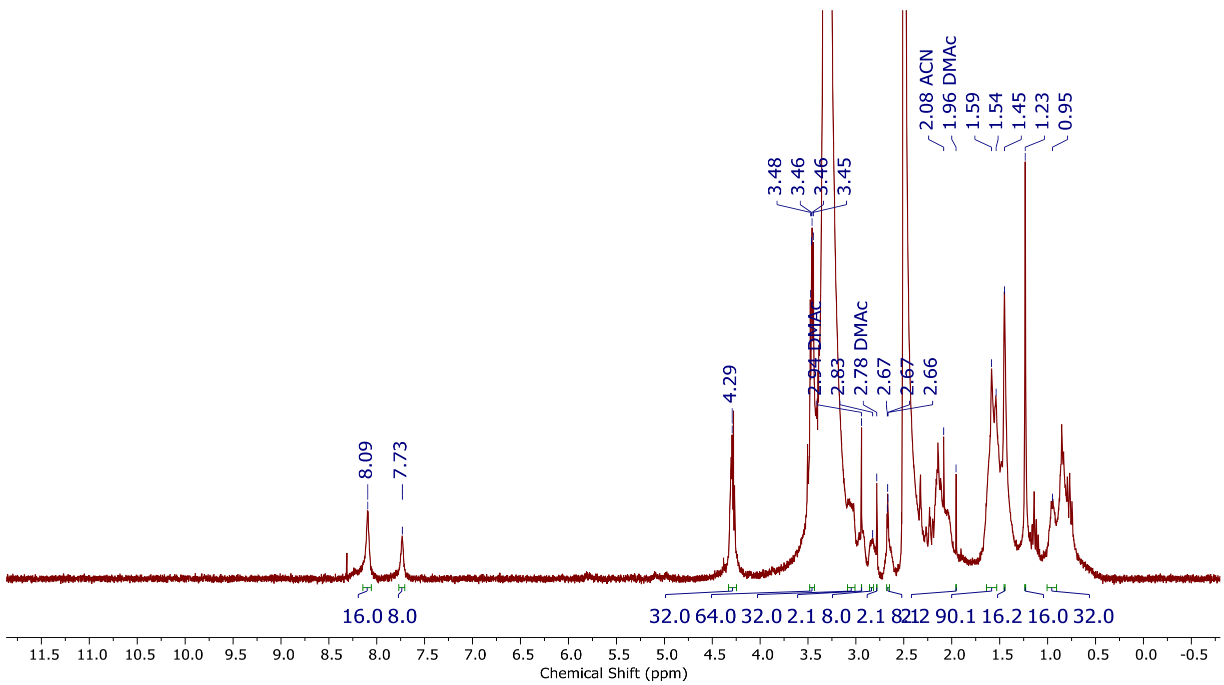

Figure S13 ^{1}H NMR spectrum of $[(CO_3)@Ag_{20}(S^tBu)_{10}(3,5\text{-}HOC_{10}H_{13}N_3\text{-}C_6H_3COO)_8(DMAc)_4]$ (**1d** + BCN) in DMSO-d_6

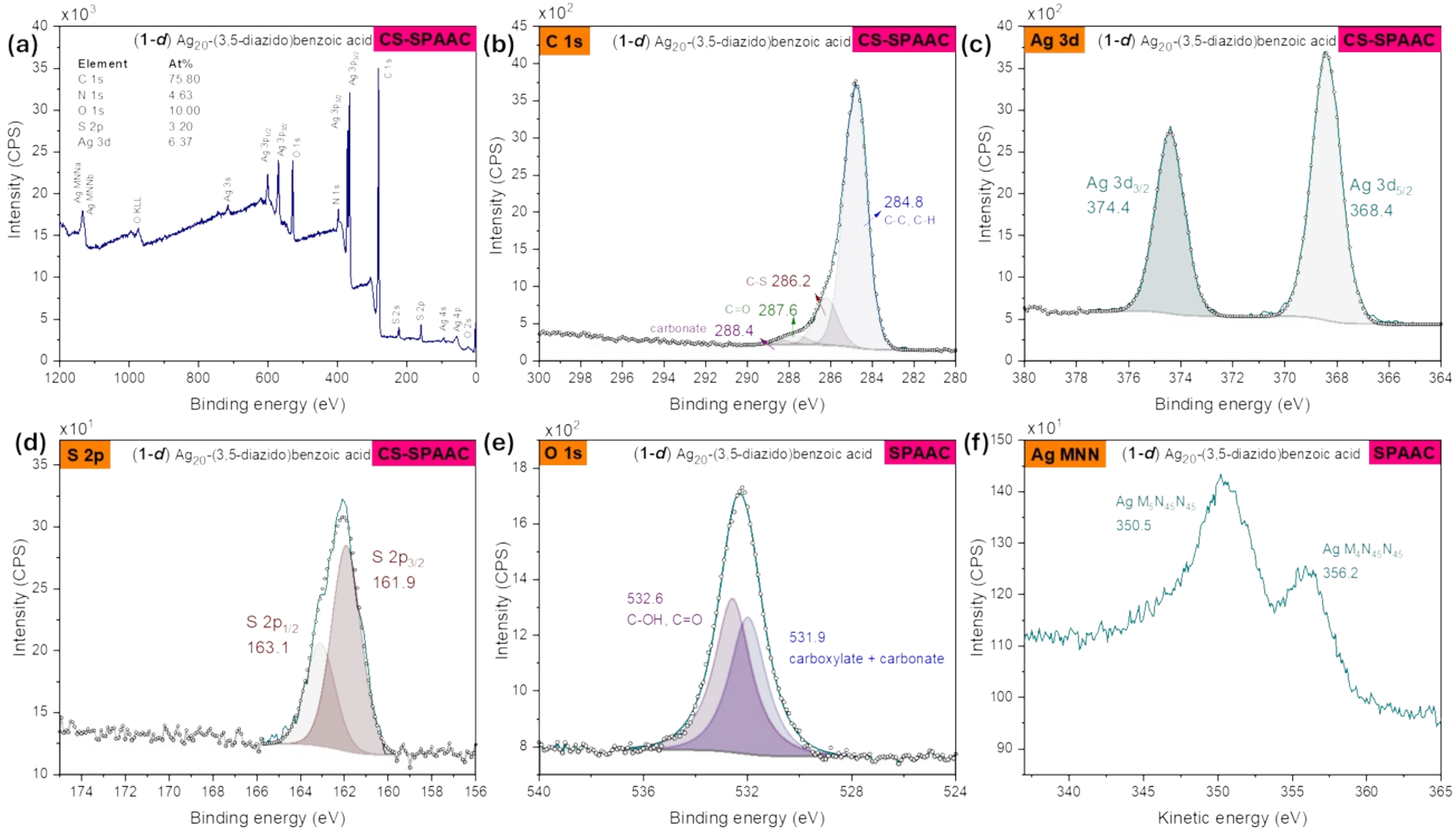

Figure S14 High resolution XPS spectra of **1** ***d*** + BCN (**1** ***d*** **SPAAC**) (a) survey spectrum (b) C 1s (c) Ag 3d (d) S 2p (e) O 1s (f) Ag MNN. C 1s was fitted with C-C 284.8 eV, C-S 286.2 eV, carbonate 288.4 and C=O 287.6 eV. Ag 3d was fitted with two spin-orbit components Ag $3d_{3/2}$ and Ag $3d_{5/2}$. S 2p was fitted considering S $2p_{1/2}$ and S $2p_{3/2}$ spin-orbit components with fixed doublet separation of 1.18eV and constrained area ratio; S $2p_{3/2}$ Ag-S 161.9 eV. O 1s was fitted with two components: 531.9 corresponding to carboxylate, and 532.6 eV carbonate + OH + C=O.

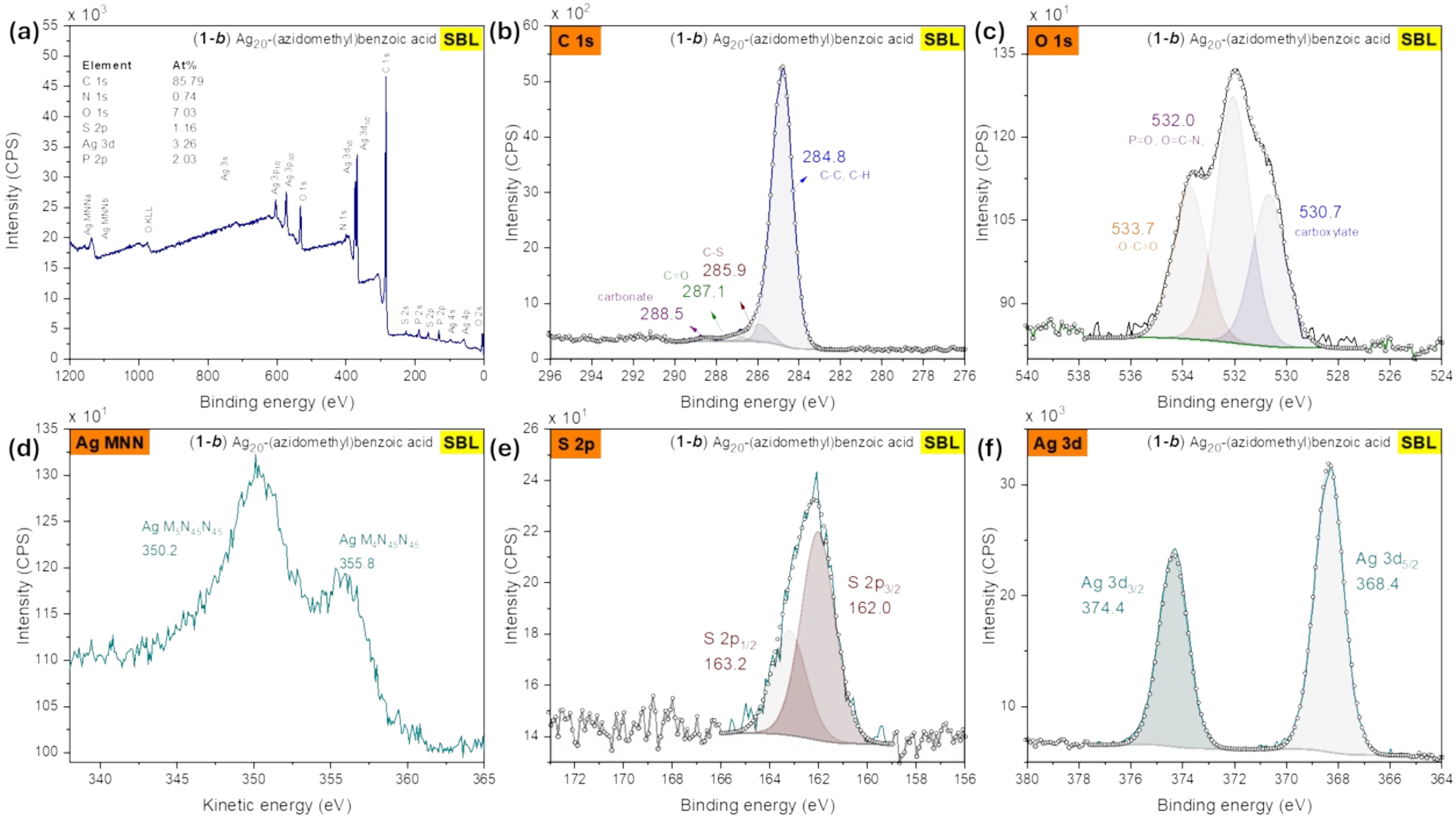

Figure S15 High resolution XPS spectra of **1** *b* **SBL** (a) survey spectrum (b) C 1s (c) O 1s (d) Ag MNN (e) S 2p (f) Ag 3d. C 1s was fitted with C-C 284.8 eV, C-S 285.9 eV, carbonate 288.5 and C=O 287.1 eV. Ag 3d was fitted with two spin-orbit components Ag $3d_{3/2}$ and Ag $3d_{5/2}$. S 2p was fitted considering S $2p_{1/2}$ and S $2p_{3/2}$ spin-orbit components with fixed doublet separation of 1.18eV and constrained area ratio; S $2p_{3/2}$ Ag-S 162.0 eV. O 1s was fitted with three components: 530.7 corresponding to carboxylate, 532.0 P=O + amide and 533.7 eV ester.

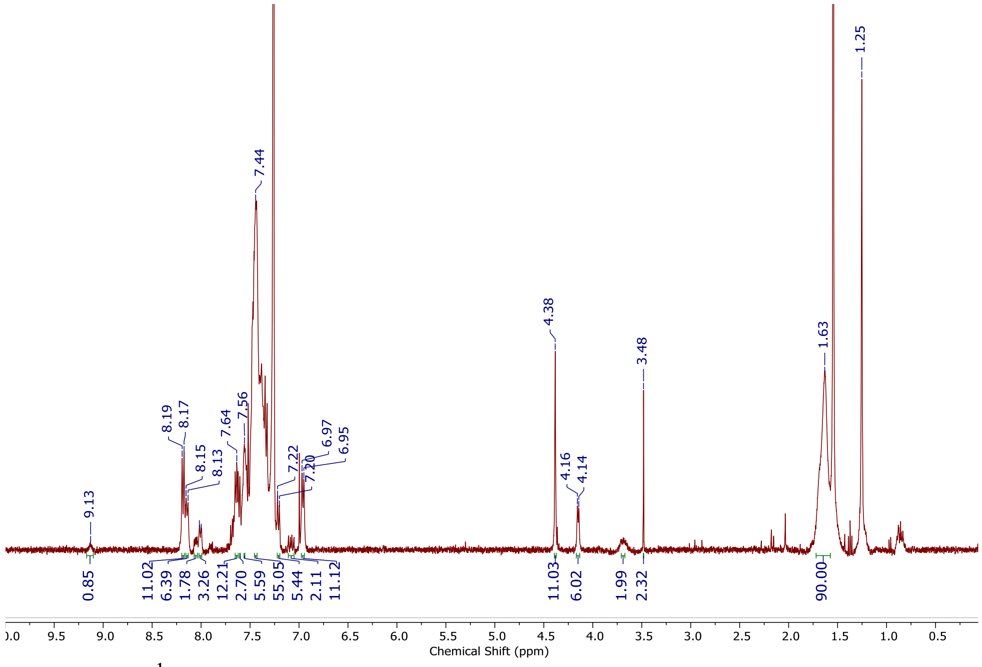

Figure S16 ^{1}H NMR spectrum of **1** ***b*** **SBL** collected in $CDCl_3$

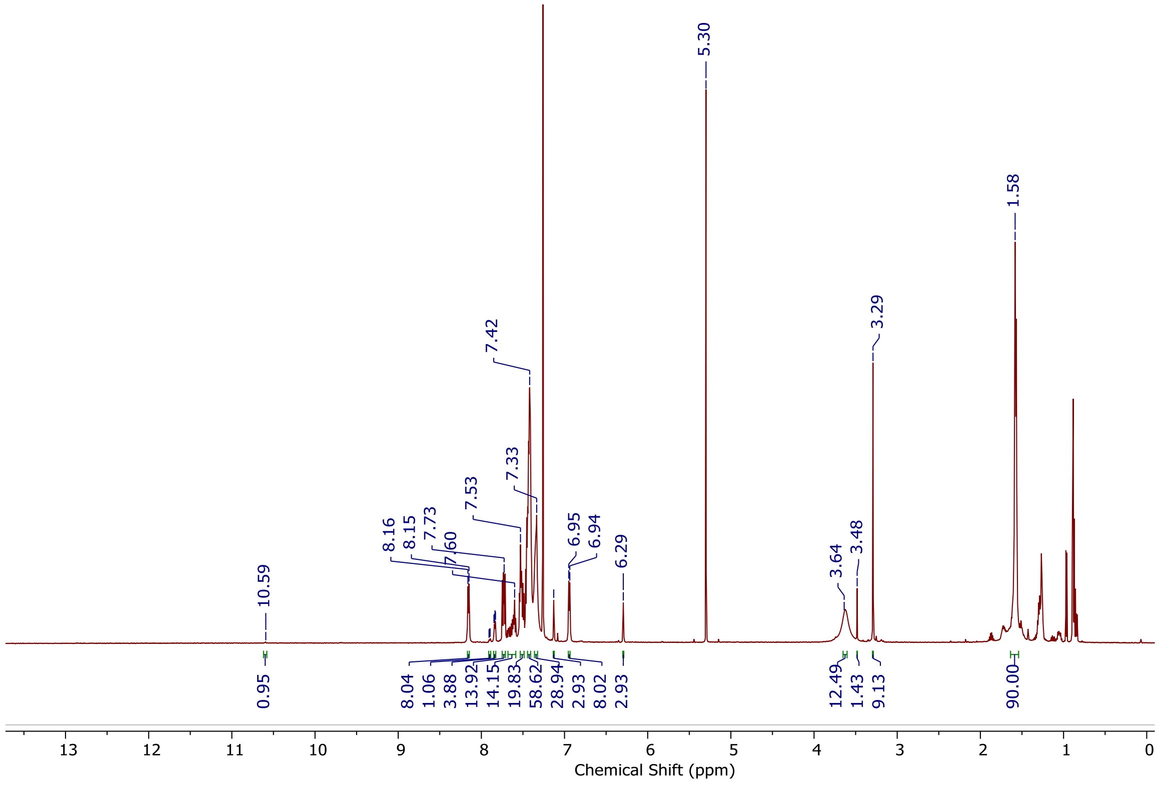

Figure S17 ^{1}H NMR spectrum of **1** ***d*** **SBL** collected in $CDCl_3$

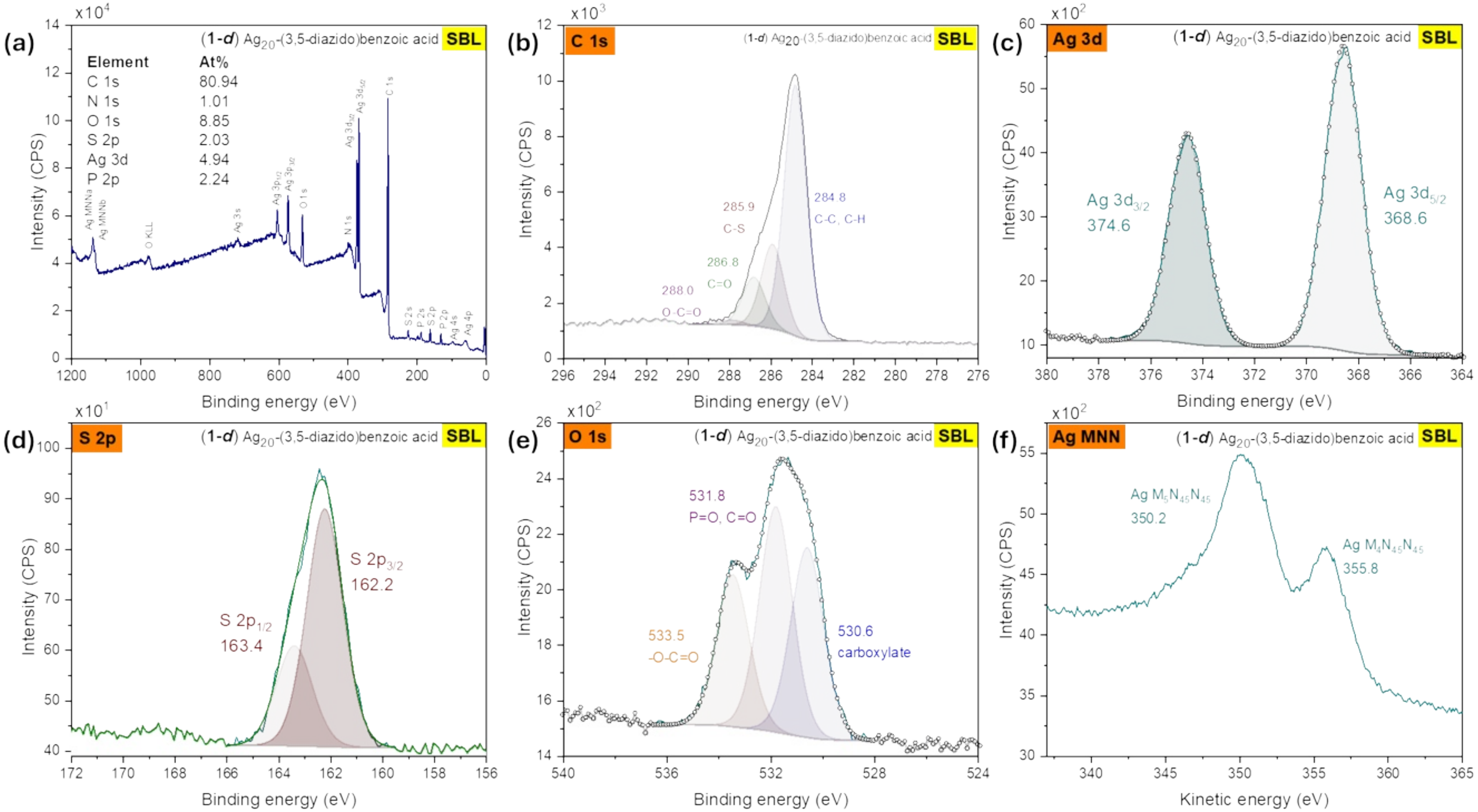

Figure S18 High resolution XPS spectra of **1** ***d*** **SBL** (a) survey spectrum (b) C 1s (c) Ag 3d (d) S 2p (e) O 1s (f) Ag MNN. C 1s was fitted with C-C 284.8 eV, C-S 285.9 eV, carbonate 288.5 and C=O 287.1 eV. Ag 3d was fitted with two spin-orbit components Ag $3d_{3/2}$ and Ag $3d_{5/2}$. S 2p was fitted considering S $2p_{1/2}$ and S $2p_{3/2}$ spin-orbit components with fixed doublet separation of 1.18eV and constrained area ratio; S $2p_{3/2}$ Ag-S 162.0 eV. O 1s was fitted with three components: 530.7 corresponding to carboxylate, 532.0 P=O + amide and 533.7 eV ester.

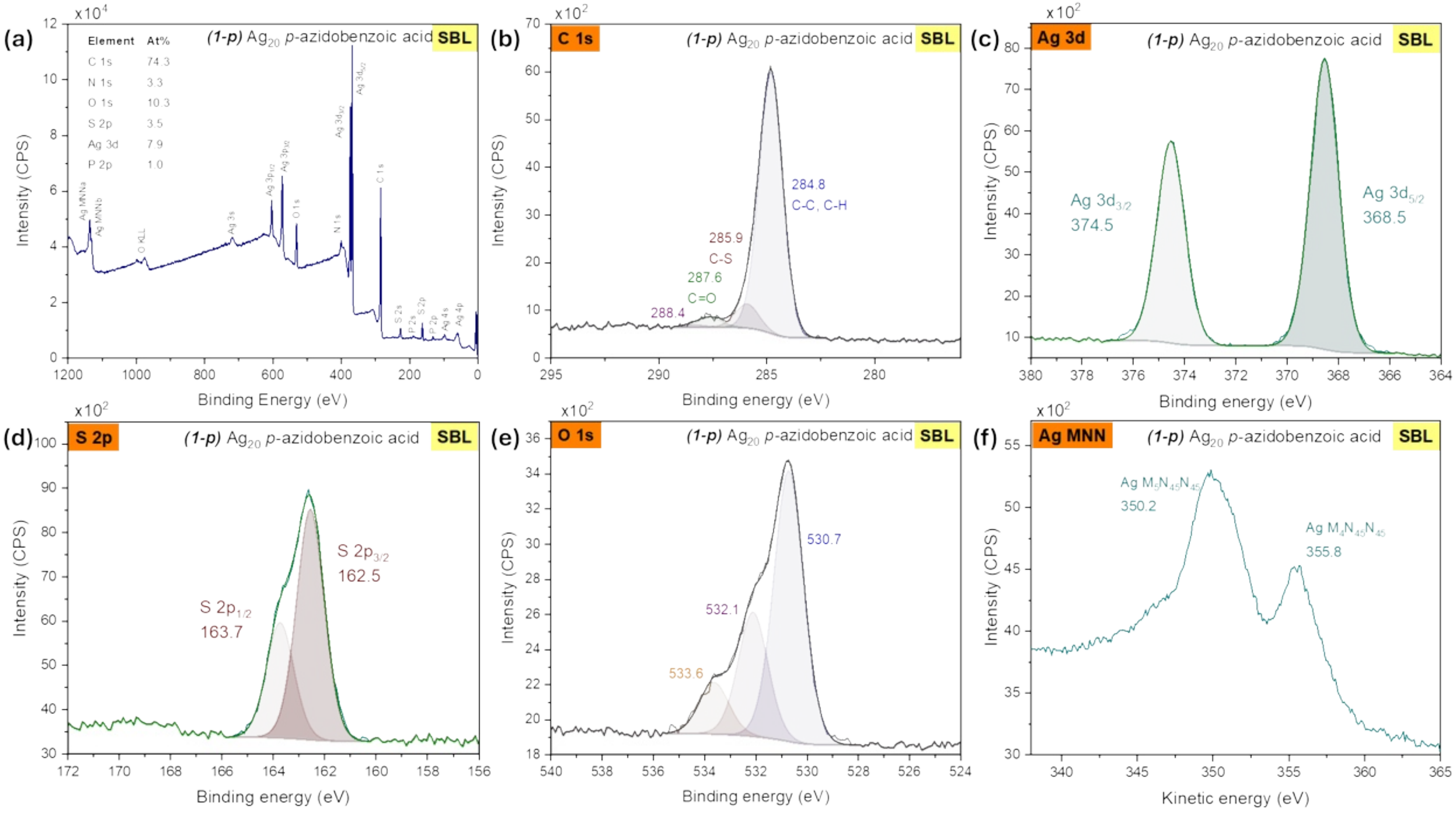

Figure S19 High resolution XPS spectra of **1** ***p*** **SBL** (a) survey spectrum (b) C 1s (c) Ag 3d (d) S 2p (e) O 1s (f) Ag MNN. C 1s was fitted with C-C 284.8 eV, C-S 285.9 eV, carbonate 288.4 and C=O 287.6 eV. Ag 3d was fitted with two spin-orbit components Ag 3d$_{3/2}$ and Ag 3d$_{5/2}$. S 2p was fitted considering S 2p$_{1/2}$ and S 2p$_{3/2}$ spin-orbit components with fixed doublet separation of 1.18eV and constrained area ratio; S 2p$_{3/2}$ Ag-S 162.5 eV. O 1s was fitted with three components: 530.7 corresponding to carboxylate, 532.1 P=O + amide and 533.6 eV ester.

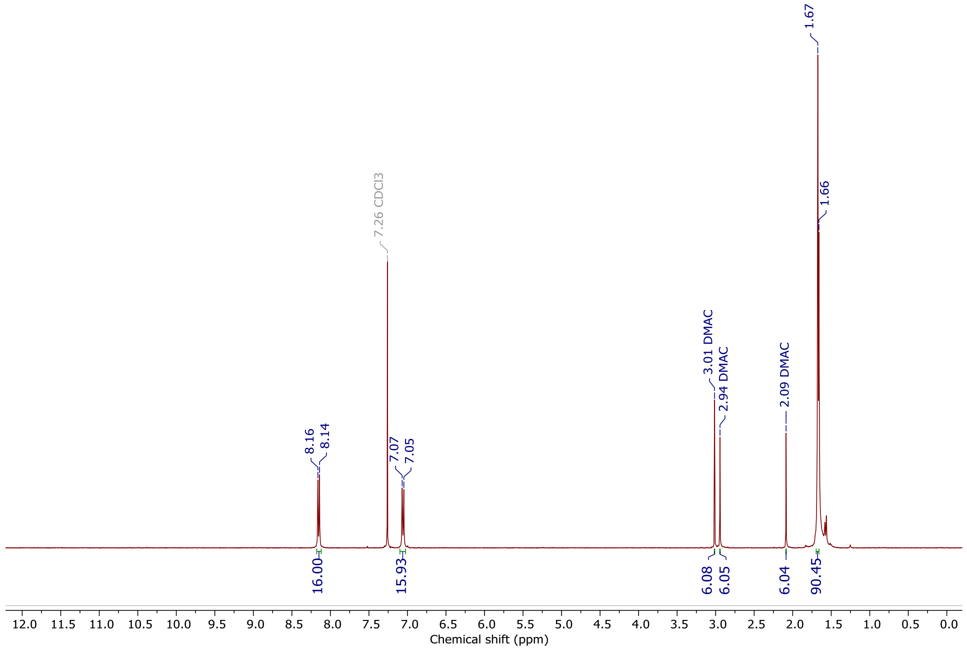

Figure S20 ^{1}H NMR spectrum of **1** ***p*** collected in $CDCl_3$

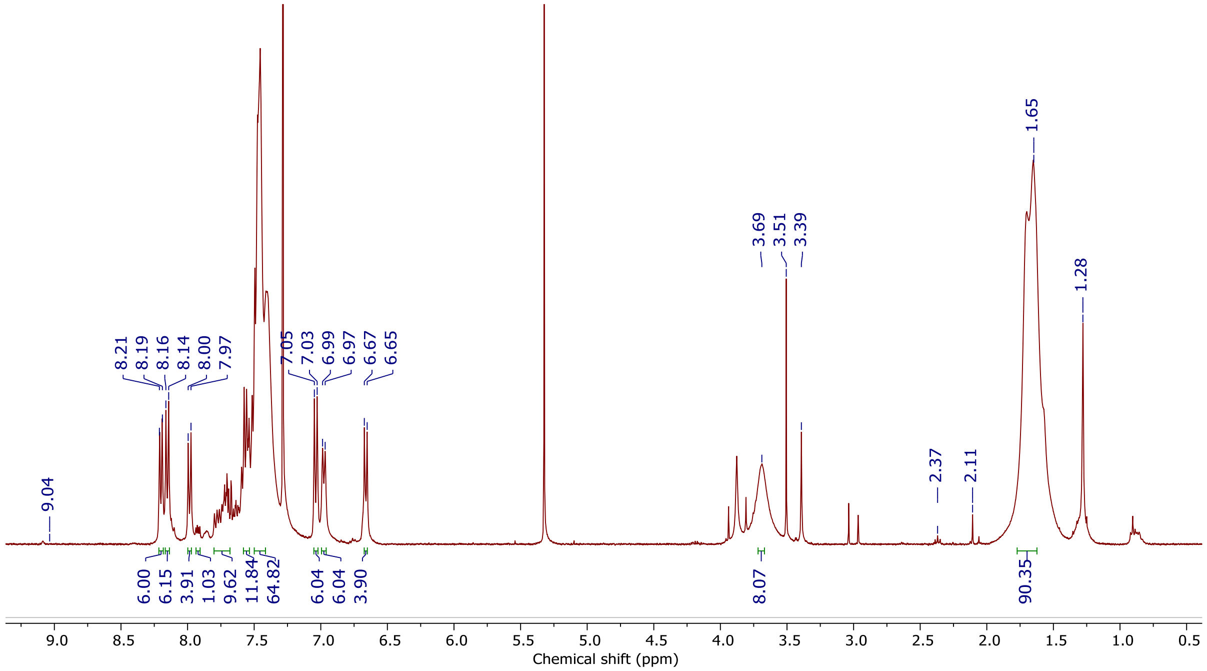

Figure S21^{1}H NMR spectrum of **1** ***p*** **SBL** collected in CDCl3

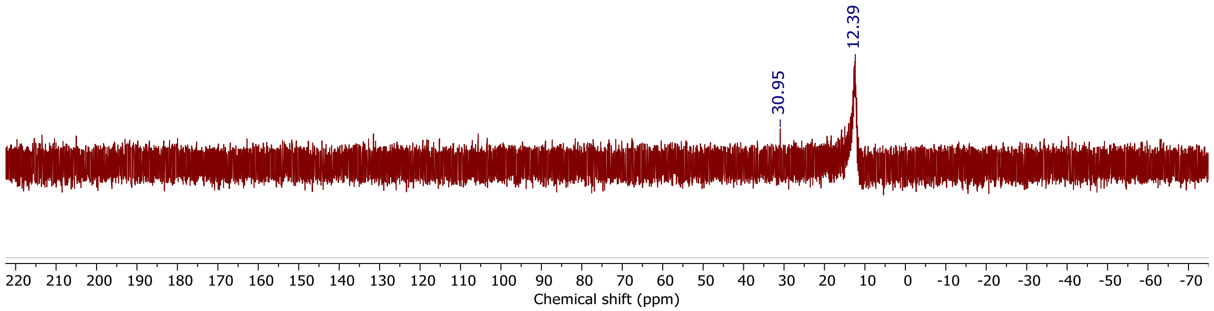

Figure S22 $^{31}P\{^{1}H\}$ NMR spectrum of parent cluster-SBL collected in $CDCl_3$ *Parent cluster in this spectrum is referred to a Ag_{20} cluster synthesized with benzoic acid, azide moiety is not present.

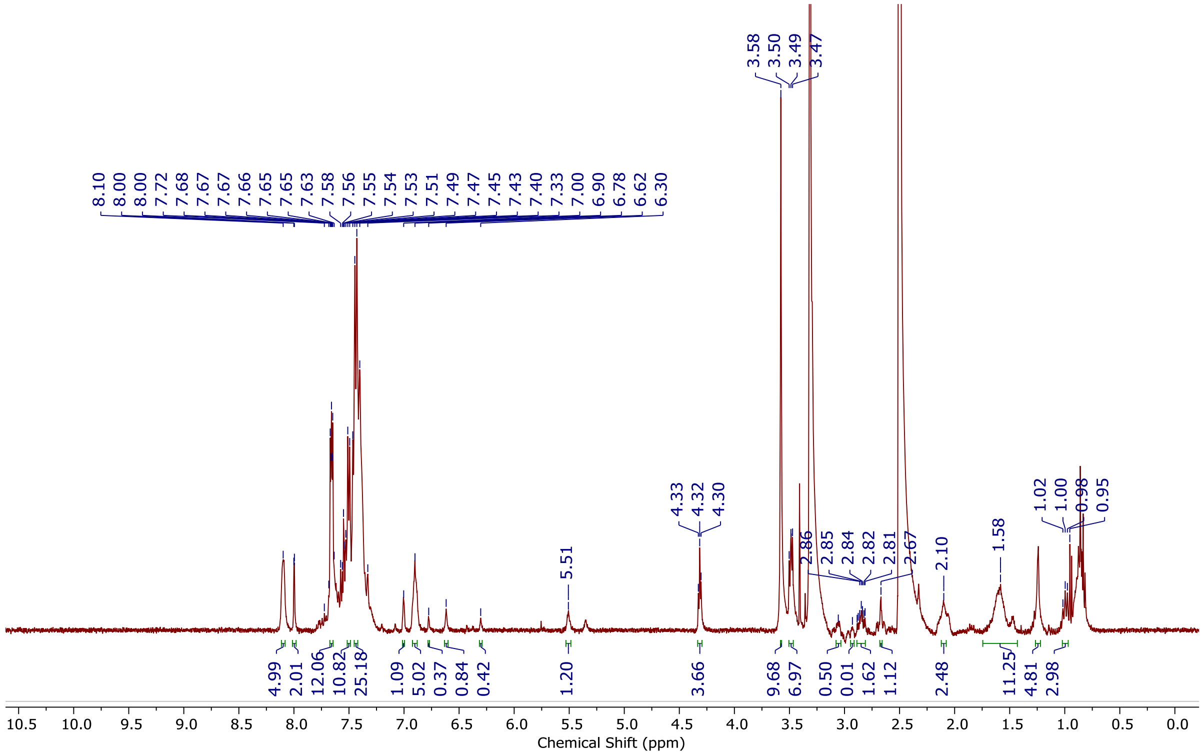

Figure S23 ^{1}H NMR spectrum of **1** ***d*** **SBL** **SPAAC** in DMSO-d_6.

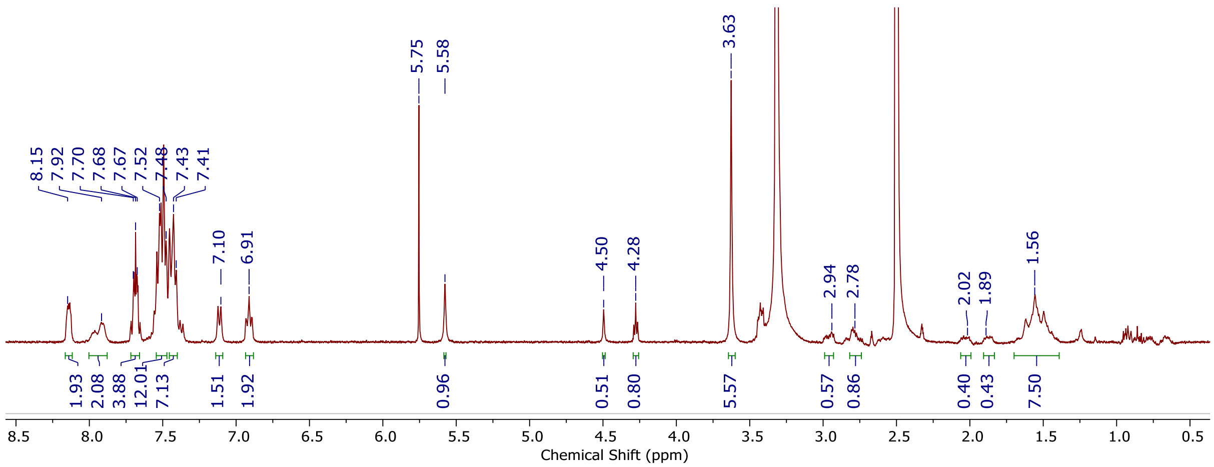

Figure S24 ^{1}H NMR spectrum of **1** ***b*** **SBL SPAAC** in DMSO-d_6

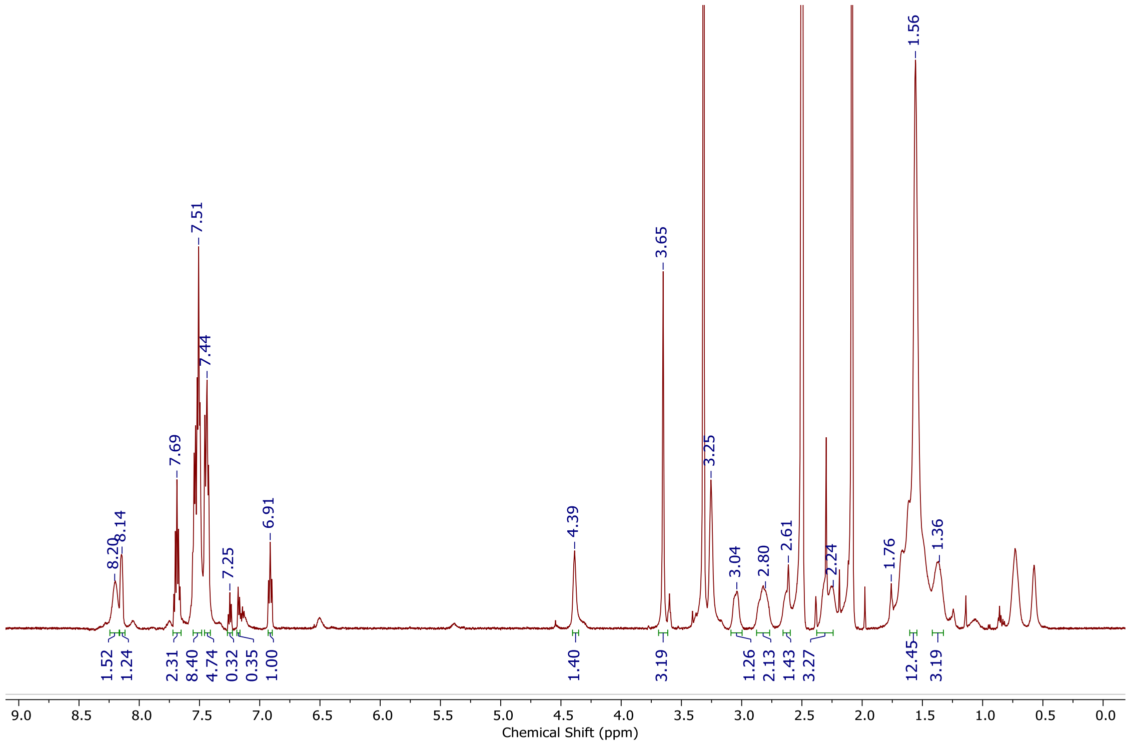

Figure S25 ^{1}H NMR spectrum of **1** ***p*** **SBL SPAAC** collected in DMSO-d_6

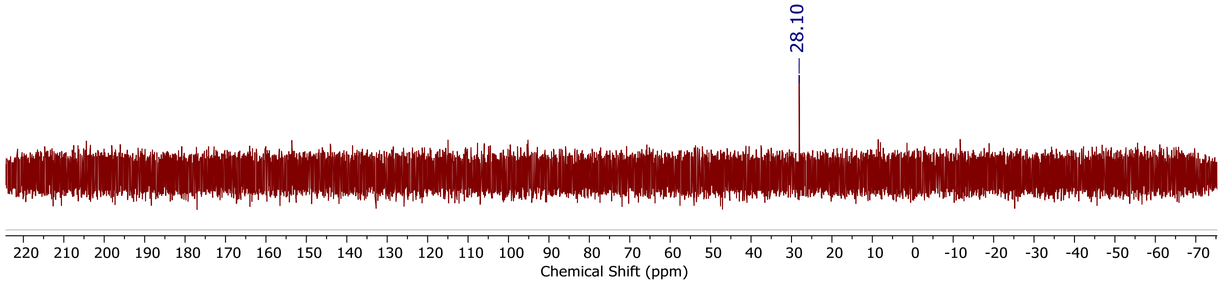

Figure S26 $^{31}P\{^{1}H\}$ NMR spectrum of **1** ***d*** **SBL SPAAC** in DMSO-d_6.

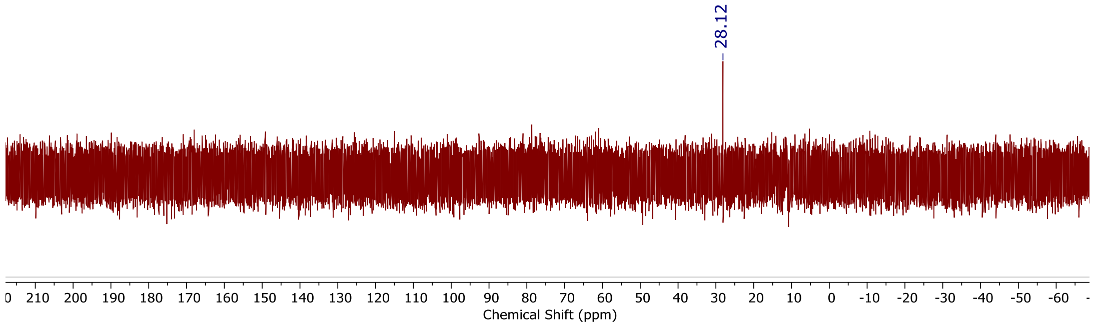

Figure S27 $^{31}P\{^{1}H\}$ NMR spectrum of **1** ***b*** **SBL SPAAC** in DMSO-d_6.

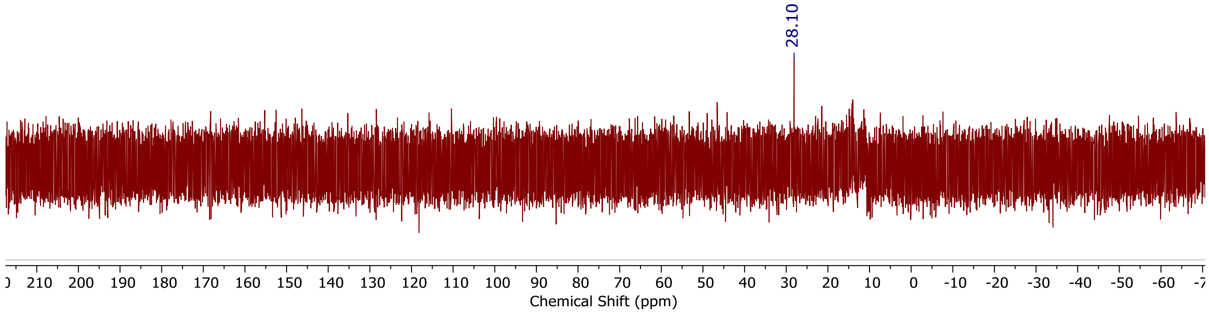

Figure S28 $^{31}P\{^{1}H\}$ NMR spectrum of **1** ***p*** **SBL SPAAC** collected in DMSO-d_6

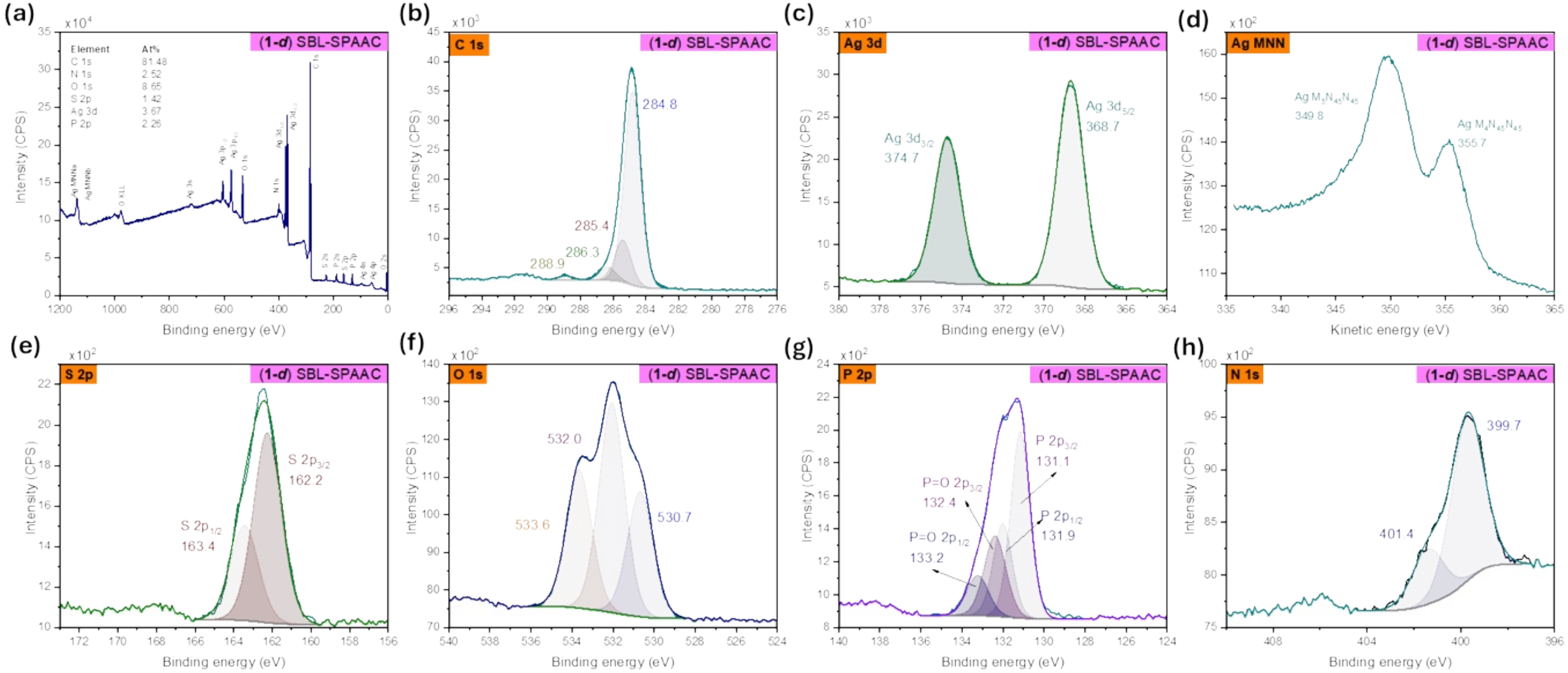

Figure S29 High resolution XPS spectra of **1** ***d*** **SBL SPAAC** (a) survey spectrum (b) C 1s (c) Ag 3d (d) Ag MNN (e) S 2p (f) O 1s (g) P 2p (h) N 1s. C 1s was fitted with C-C 284.8 eV, C-S 285.4 eV, C-OH 286.3 eV, and carbonate 288.9 eV. Ag 3d was fitted with two spin-orbit components Ag $3d_{3/2}$ and Ag $3d_{5/2}$. S 2p was fitted considering the two spin-orbit components S $2p_{1/2}$ and S $2p_{3/2}$ with a doublet separation of 1.18eV and constrained area ratio, S $2p_{3/2}$ Ag-S 162.2 eV. O 1s fitted with three components 530.7 eV assigned to carboxylate, 532.0 eV P=O + amide and 533.6 eV ester. P 2p was fitted considering a doublet separation P $2p_{3/2}$- P $2p_{1/2}$ of 0.86 eV and constrained area ratio, P $2p_{3/2}$ P=O 132.4 eV ±0.4 eV. N 1s was fitted with N-C 399.7 eV and N=N 401.4 eV.

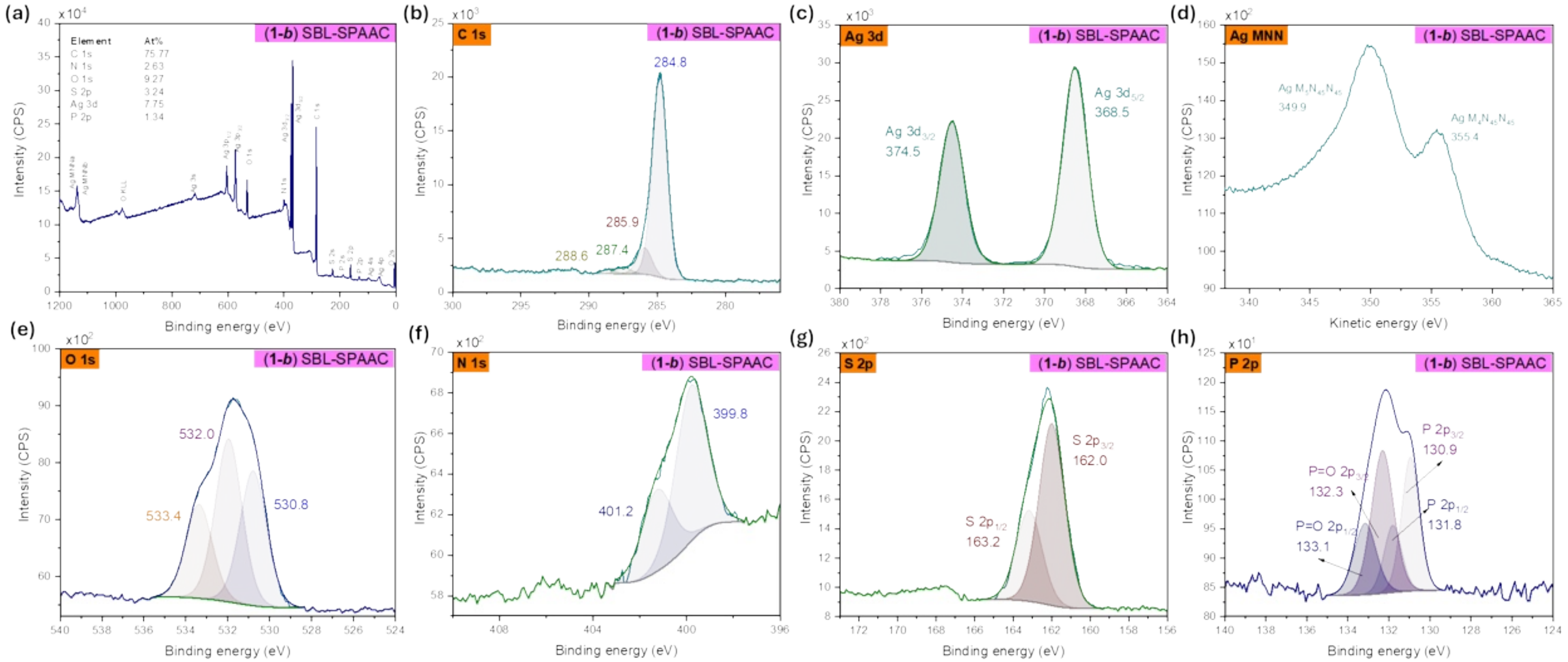

Figure S30 High resolution XPS spectra of **1** ***b*** **SBL SPAAC** (a) survey spectrum (b) C 1s (c) Ag 3d (d) Ag MNN (e) O 1s (f) N 1s (g) S 2p (h) P 2p. C 1s was fitted with C-C 284.8 eV, C-S 285.9 eV, C=O 287.4 eV, and carbonate 288.6 eV. Ag 3d was fitted with two spin-orbit components Ag $3d_{3/2}$ and Ag $3d_{5/2}$. S 2p was fitted considering the two spin-orbit components S $2p_{1/2}$ and S $2p_{3/2}$ with a doublet separation of 1.18eV and constrained area ratio, S $2p_{3/2}$ Ag-S 162.0 eV. O 1s fitted with three components 530.8 eV assigned to carboxylate, 532.0 eV P=O + amide and 533.4 eV ester. P 2p was fitted considering a doublet separation P $2p_{3/2}$- P $2p_{1/2}$ of 0.86 eV and constrained area ratio, P $2p_{3/2}$ P=O 132.6 eV ±0.4 eV. N 1s was fitted with N-C 399.7 eV and N=N 401.2 eV.

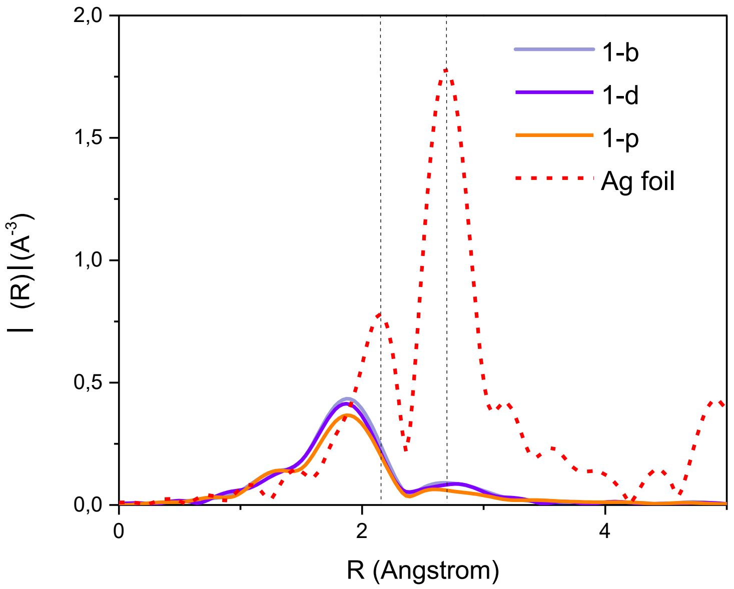

Figure S31 Fourier transformed Ag K-edge EXAFS spectra, comparison of intensity between **1 *b***, **1 *d***, **1 *p*** and Ag foil

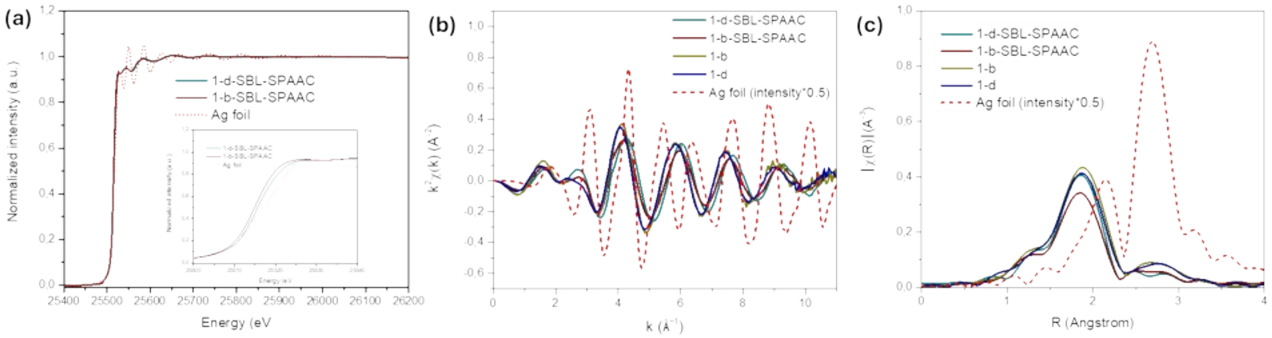

Figure S32 Data for **1 *b* SBL SPAAC** and **1 *d* SBL SPAAC** (a) Ag K-edge XAS spectra with an inset of the XANES region (b) Ag K-edge FT-EXAFS shown in K-space and (c) R-space

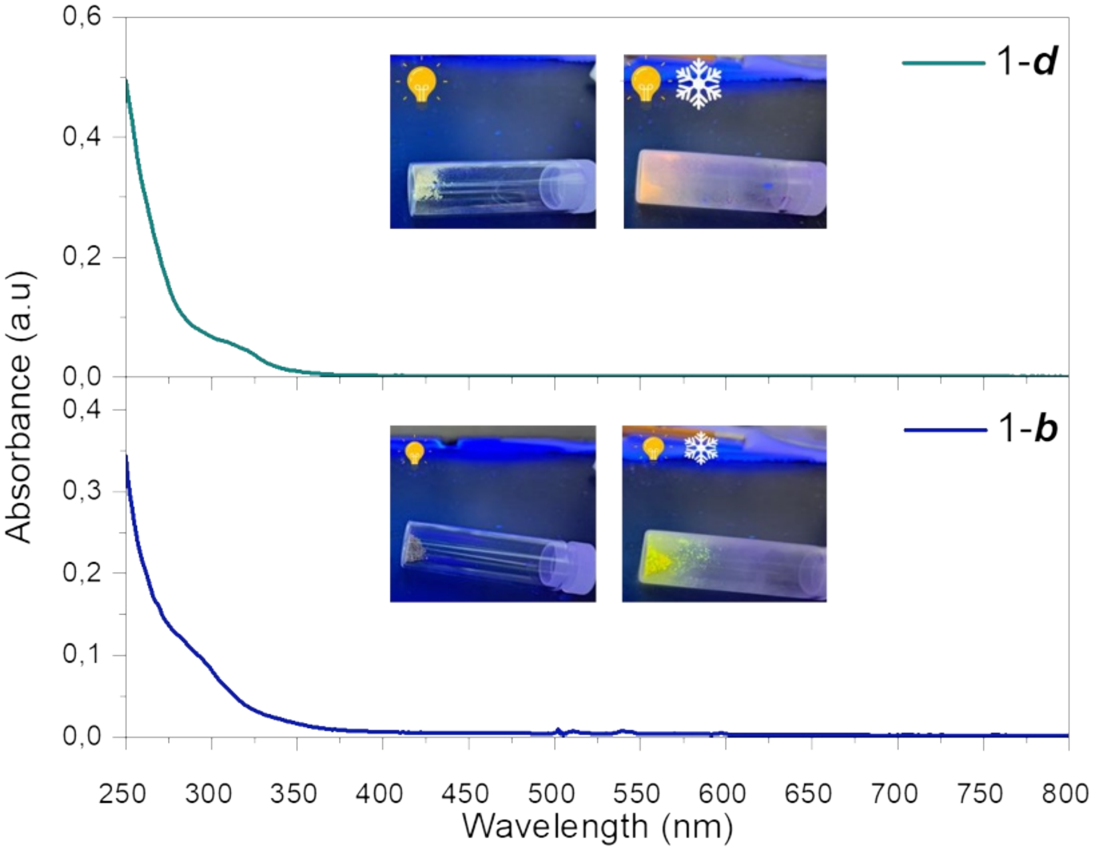

Figure S 33 UV-Vis absorbance spectra of a) 0.0016 mM of 1 *d* solution in DCM, inset shows photos of the solid NC under UV-light at room temperature and cooled with liquid nitrogen b) 0.0022 mM of 1 *b* solution in DCM, inset shows photos of the solid NC under UV-light at room temperature and cooled with liquid nitrogen

Table S 1 Summary of peak fragments found in ESI-MS spectra of clicked products.

1-d-SBL		
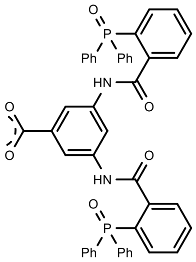	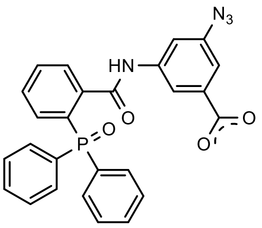	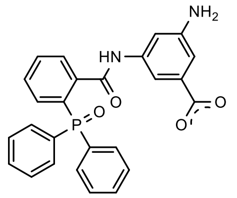
$C_{45}H_{33}O_6N_2P$	$C_{26}H_{18}O_4N_4P$	$C_{26}H_{20}O_4N_2P$
Peak found m/z	Peak found m/z 481.10	Peak found m/z 455
1-d-SPAAC		
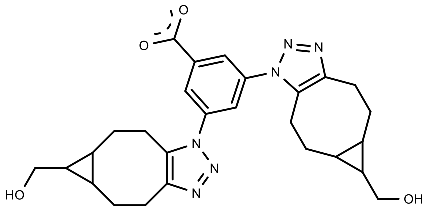		
$C_{27}H_{31}O_4N_6$		
Peak found m/z		
1-d-SBL-SPAAC		

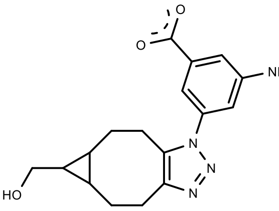	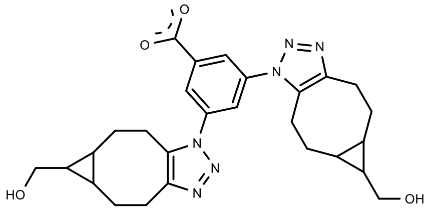	
$C_{17}H_{19}O_3N_4$	$C_{27}H_{31}O_4N_6$	
Peak found m/z	Peak found m/z	
1-b-SBL-SPAAC		
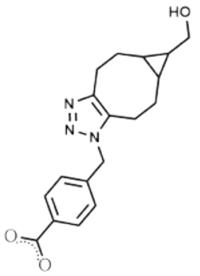	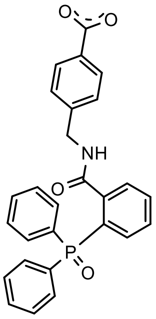	
$C_{18}H_{20}O_3N_3$	$C_{27}H_{21}O_4NP$	
Peak found m/z	Peak found m/z	
1-b-SBL		
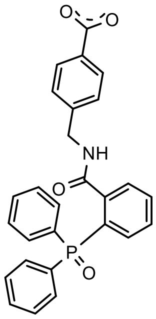	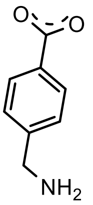	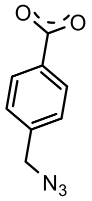
$C_{27}H_{21}O_4NP$	$C_8H_8O_2N$	$C_8H_6O_2N_3$
Peak found m/z	Peak found m/z	Peak found m

Crystallographic Information

Data collection and processing. The samples (**1** ***b*** and **1** ***d***) were mounted on a Mitegen polyimide micromount with a small amount of Paratone N oil. All X-ray measurements were made on a Bruker Kappa Axis Apex2 diffractometer at a temperature of 110 K.

It was apparent from the initial indexing that the sample crystal **1** ***d*** was a non-merohedric twin and was indexed to two domains (*vide infra*). The unit cell dimensions were determined from a symmetry constrained fit of 9966 reflecti
strategy was a number of ω and φ scans whic
integration was performed using SAINT.[S1] The resulting raw data was scaled and absorption corrected using a multi-scan averaging of symmetry equivalent data using TWINABS.[S2]
The unit cell dimensions of **1** ***b*** were determined from a symmetry constrained fit of 9911 reflections with 5.5° < 2θ < 64.82°. The dat
which collected data up to 65.464° (2θ). The[S1]
The resulting raw data was scaled and absorption corrected using a multi-scan averaging of symmetry equivalent data using SADABS.[S2]

Analysis of Twinning (**1** *d*). The diffraction pattern was indexed to two domains. The metrical relations between the domains are given below:

```
=====================================================================
===========
Solution number :   1
---------------------
New Cell: a=15.3565 b=15.5344 c=19.7274 alpha=105.028 beta=109.577 gamma=90.647

Figure of Merit (0=ideal)         :   0.00
Rotation angle  (degrees)         :-179.883
Rotation vector (laboratory)      :  -0.6750  0.6530  0.3435
Rotation vector (reciprocal cell) :  -1.00    1.00    0.00
Rotation vector (direct cell)     : -14.00   12.93   -1.00

Superposition matrix              : H' = +0.040 * H -0.959 * K +0.075 * L
                                    K' = -1.040 * H -0.039 * K -0.073 * L
                                    L' = -0.004 * H           -1.001 * L
=====================================================================
===========
```

Structure Solution and Refinement. The structure was solved by using a dual space methodology using the SHELXT program.[S3] Most non-hydrogen atoms were obtained from the initial solution. The remaining atomic positions were obtained from subsequent difference Fourier maps. The hydrogen atoms were introduced at idealized positions and were allowed to ride on the parent atom.

The structure **1** *d* exhibits a variety of disorders as well as highly disordered solvent. The structure was subjected to a solvent masking procedure as implemented by the SQUEEZE routine in PLATON.[S4] The disorders of **1** *d* were as follows: 1) The carbonate template anion was disordered over a crystallographic inversion centre. 2) Atom AG4 was disordered over two positions separated by 0.61 Å; the occupancy factor for the primary position refined to a value of 0.890(13). 3) The methyl groups of the *t*-butyl group attached to atom S5 were disordered over two orientations. The occupancy factor for the major rotamer refined to a value of 0.754(9). 4) The coordinated acetamide molecule containing atoms O2S, C5S, C6S, N2S, C7S, and C8S was disordered over two positions. The occupancy factor for the major component for this disorder refined to a value of 0.573(13). The structural model was fit to the data from both twin components using full matrix least-squares based on F^2. The twin fraction refined to a value of 0.4139(6).

The calculated structure factors included corrections for anomalous dispersion from the usual tabulation. The structure was refined using the SHELXL program from the SHELX suite of crystallographic software.[S3]

Structure 1 ***b:*** As typical for this class of compound the template CO_3^{2-} anion was disordered across a crystallographic centre of symmetry. The nitrate anion was disordered over two orientations. The occupancy of the primary oriented refined to a value of 0.556(6). Silver atoms AG6, AG7, and AG8 were disordered over two sites with the occupancy factor for the primary site refining to a value of 0.7041(16). Silver atom AG10 (disorder site AG11) was disordered over two sites. The occupancy factor refine
disordered over two orientations and the azide group for this ligand was disordered over three orientations. This disorder was modelled using three different occupancy factors. The occupancy factors refined to values 0.485(14), 0.367(14), and 0.147(4). In addition, the asymmetric unit contained regions of disordered solvents for which a sensible disorder model

could not be found. As a consequence, the structure was subjected to the SQUEEZE procedure as implemented by the PLATON program.[S4] The structural model was fit to the data using full matrix least-squares based on F^2. The calculated structure factors included corrections for anomalous dispersion from the usual tabulation. The structure was refined using the SHELXL program from the SHELX suite of crystallographic software.[S3]

Graphic plots were produced using the Mercury program.[S5] Additional information and other relevant literature references can be found in the reference section of this website (http://xray.chem.uwo.ca).

Table S2 Summary of Crystal Data for **1** ***d*** and **1** ***b***

Formula	$C_{113}H_{150}Ag_{20}N_{52}O_{23}S_{10}$ (1–d)	$C_{105}H_{162}Ag_{20}N_{24}O_{25}S_{10}$ (1–b)
Formula Weiggh/tm)	5082.84	4638.58
Crystal Dimensi (mm)	0.276 × 0.202 ×	0.337 × 0.182
Crystal and Hab	colourless prism	colourless pri
Crystal	triclinic	triclinic
Space G	P-1	P-1
Tempera K	110	110
, Å	15.357(4)	14.992(8)
, Å	15.534(4)	16.135(8)
, Å	19.727(5)	17.859(9)
a, °	105.028(8)	91.739(11)
b, °	109.577(8)	97.931(11)
g, °	90.647(7)	113.83(2)
V, ^{3} Å	4257.4(19)	3896(3)
Number reflect determi unit ce	9966	9911
Min and 2q for determi °	5.7, 52.68	5.5, 64.82
Z	1	1
F(000)	2476	2260

rg ( ) cm	1.983	1.977
l, Å, (	0.71073	0.71073
m, cm^{-1} ()	2.435	2.647
Diffrac Type	Bruker Kappa Axi	Bruker Kappa A
Scan Ty	phi and omega sc	φ and ω scans
Max 2q data collect	60.404	65.464
Measure fractio	0.999	0.998
Number reflect measure	44905	284922
Unique reflect measure	44905	28505
R_{merge}		0.0547
Number reflect include refinem	44905	28505
Cut off Thresho Express	I > 2sigma(I)	I > 2σ(I)
Structu refined	full matrix least-squares on F^2	full matrix least-squares on F^2
Weighti Scheme	w=1/[σ^2(Fo^2)+(0.0850P)^2+0.67[illegible] P] where P=(Fo^2+2Fc^2)/3	w=1/[σ^2(Fo^2)+(0.0313P)^2+[illegible] P] where P=(Fo^2+2Fc^2)/3
Number paramet least-squa	1060	988
R_1	0.0572	0.0419
wR_2	0.1339	0.0958
R_1 (all	0.0869	0.0606
wR_2 (all	0.1509	0.1062
GOF	1.033	1.038
Maximum shift/e	0.001	0.002
Min & M	-1.380, 2.890	-2.047, 3.238

peak he[illegible] on fina[illegible] Map (e^-/Å)		

Where:

$R_1 = |\,|F_o| - |F_c|\,| / |F_o|$

$wR_2 = [\,(\,(F_o^2 - F_c^2)^2\,) / (F_o^4)\,]^{½}$

GOF = $[\,((F_o^2 - F_c^2)^2) / (\text{No. of reflns.} - \text{No. of params.})\,]$

References

[S1] Bruker-AXS, SAINT version 2013.8, **2013**, Bruker-AXS.

[S2] Bruker-AXS, SADABS version 2012.1, **2012**, Bruker-AXS.

[S3] G. M. Sheldrick, "Crystal structure refineme[illegible]" *Acta Crystallographica Section C* **2015**, *71*, 3–8.

[S4] A. L. Spek, "PLATON SQUEEZE: a tool for the [illegible] contribution to the calculation of structure factors." *Acta crystallographica Section C, Structural chemistry* **2015**, *71*, 9–18.

[S5] C. F. Macrae, I. J. Bruno, J. A. Chisholm, P. R. Edgington, P. McCabe, E. Pidcock, L. Rodriguez-Monge, R. Taylor, J. van de Streek, P. [illegible] new features for the visualization and investigation of c[illegible]" *Journal of Applied Crystallography* **2008**, *41*, 466–470.
